# Viability of AMURA biomarkers from single-shell diffusion MRI in clinical studies

**DOI:** 10.3389/fnins.2023.1106350

**Published:** 2023-05-10

**Authors:** Carmen Martín-Martín, Álvaro Planchuelo-Gómez, Ángel L. Guerrero, David García-Azorín, Antonio Tristán-Vega, Rodrigo de Luis-García, Santiago Aja-Fernández

**Affiliations:** ^1^Laboratorio de Procesado de Imagen (LPI), Universidad de Valladolid, Valladolid, Spain; ^2^Cardiff University Brain Research Imaging Centre (CUBRIC), School of Psychology, Cardiff University, Cardiff, United Kingdom; ^3^Headache Unit, Department of Neurology, Hospital Clínico Universitario de Valladolid, Valladolid, Spain; ^4^Department of Medicine, Universidad de Valladolid, Valladolid, Spain

**Keywords:** alternative metrics, AMURA, brain, diffusion magnetic resonance imaging, DTI, migraine

## Abstract

Diffusion Tensor Imaging (DTI) is the most employed method to assess white matter properties using quantitative parameters derived from diffusion MRI, but it presents known limitations that restrict the evaluation of complex structures. The objective of this study was to validate the reliability and robustness of complementary diffusion measures extracted with a novel approach, Apparent Measures Using Reduced Acquisitions (AMURA), with a typical diffusion MRI acquisition from a clinical context in comparison with DTI with application to clinical studies. Fifty healthy controls, 51 episodic migraine and 56 chronic migraine patients underwent single-shell diffusion MRI. Four DTI-based and eight AMURA-based parameters were compared between groups with tract-based spatial statistics to establish reference results. On the other hand, following a region-based analysis, the measures were assessed for multiple subsamples with diverse reduced sample sizes and their stability was evaluated with the coefficient of quartile variation. To assess the discrimination power of the diffusion measures, we repeated the statistical comparisons with a region-based analysis employing reduced sample sizes with diverse subsets, decreasing 10 subjects per group for consecutive reductions, and using 5,001 different random subsamples. For each sample size, the stability of the diffusion descriptors was evaluated with the coefficient of quartile variation. AMURA measures showed a greater number of statistically significant differences in the reference comparisons between episodic migraine patients and controls compared to DTI. In contrast, a higher number of differences was found with DTI parameters compared to AMURA in the comparisons between both migraine groups. Regarding the assessments reducing the sample size, the AMURA parameters showed a more stable behavior than DTI, showing a lower decrease for each reduced sample size or a higher number of regions with significant differences. However, most AMURA parameters showed lower stability in relation to higher coefficient of quartile variation values than the DTI descriptors, although two AMURA measures showed similar values to DTI. For the synthetic signals, there were AMURA measures with similar quantification to DTI, while other showed similar behavior. These findings suggest that AMURA presents favorable characteristics to identify differences of specific microstructural properties between clinical groups in regions with complex fiber architecture and lower dependency on the sample size or assessing technique than DTI.

## 1. Introduction

Diffusion Magnetic Resonance Imaging (dMRI) is an imaging modality employed to assess diverse *in vivo* physiological and pathological conditions of the human body in clinical studies. It has been widely used in the study of the brain and neurological disorders (Rovaris et al., 2005; Goveas et al., 2015; Galbán et al., 2017; Mekkaoui et al., 2017). It allows the characterization of the diffusivity of water molecules within the tissue, providing information about the microscopic configuration and structural connectivity of the brain, especially inside the white matter (WM).

The most relevant feature of dMRI is its ability to measure directional variance, i.e., anisotropy, which, inside the brain, is related to structural connectivity between areas. The most common methodology to estimate the anisotropy is via the diffusion tensor (DT) (Basser et al., 1994; Westin et al., 2002).

In order to use it in clinical studies, the information provided by the DT must be translated into some scalar measures that describe different features of diffusion within every voxel. That way, metrics like fractional anisotropy (FA) were defined and widely employed to characterize damaged tissues in multiple neurological and psychiatric disorders (Kochunov et al., 2012; Bette et al., 2016; Mole et al., 2016; Herbert et al., 2018). However, from the early stages of DT imaging (DTI), it was clear that the Gaussian assumption oversimplifies the diffusion process.

In the past few decades, many techniques have been proposed to overcome the limitations of DTI, usually requiring the acquisition of larger amounts of diffusion data (Assemlal et al., 2011; Novikov et al., 2019). Most of these techniques rely on the estimation of more advanced diffusion descriptors, such as the Ensemble Average diffusion Propagator (EAP), which is the probability density function of the motion of the water molecules within a voxel (Wedeen et al., 2005; Özarslan et al., 2013; Tristán-Vega and Aja-Fernández, 2021).

A complete analysis of the EAP requires many diffusion-weighted images (DWI) with several (moderate to high) *b*-values in a multi-shell acquisition. The information provided by the EAP is usually adapted to scalar measures that describe different aspects of diffusion. The most frequently employed measures are the return-to-origin probabilities (RTOP), return-to-plane probabilities (RTPP), return-to-axis probabilities (RTAP) and the propagator anisotropy (PA) (Wu et al., 2008; Descoteaux et al., 2011; Hosseinbor et al., 2013; Özarslan et al., 2013; Ning et al., 2015).

The accurate estimation of these measures requires the calculation of the EAP, which commonly involves: (1) long acquisition times; (2) several shells with large *b*-values, which may be difficult to acquire in many commercial MRI scanners; and (3) heavy computational burdens with very long processing times. These three issues have hindered the general adoption of EAP-related metrics in the clinical routine, despite the growing interest in the exploration of their potential applicability (Avram et al., 2016; Brusini et al., 2016; Zucchelli et al., 2016; Boscolo Galazzo et al., 2018).

To overcome these limitations and facilitate the widespread use of advanced diffusion metrics in clinical studies, a new approach called Apparent Measures Using Reduced Acquisitions (AMURA) has been recently proposed (Aja-Fernández et al., 2020, 2021, 2022). The method allows the estimation of diffusion measures such as RTOP, RTAP, and PA, while reducing the number of necessary samples and the computational cost. AMURA can mimic the sensitivity of EAP-based measures to microstructural changes when only a small number of shells (even one) is available. To do so, AMURA assumes a prior model for the behavior of the radial q-space instead of trying to numerically describe it, yielding simplified expressions that can be computed easily even from single-shell acquisitions.

One additional advantage of AMURA is that it can be easily integrated into the processing pipeline of current existing single-shell dMRI protocols and databases to unveil anatomical details that may remain hidden in traditional DT-based studies. AMURA has proved its potential in some exploratory studies with clinical data focusing on Parkinson's disease and Mild Cognitive Impairment (Aja-Fernández et al., 2020, 2021), as well a recent clinical study on migraine (Planchuelo-Gómez et al., 2020c).

In this work, we aim to assess the viability of different diffusion descriptors extracted with AMURA for the study of a neurological disorder in DTI-type datasets. Note that, initially, AMURA was designed to work with *b*-values over 2,000 s/mm^2^, since the effects measured with RTOP, RTPP, and RTAP were better showed at higher values of b. However, results in clinical data have shown its potential at lower *b*-values (Aja-Fernández et al., 2022). Thus, we will explore the viability of these technique to model DTI-type acquisitions, i.e., dMRI datasets acquired with those protocols usually employed for the estimation of DTI and its derived parameters, such as fractional anisotropy (FA) or mean diffusivity (MD). These acquisitions are commonly single-shell, and only include one non-zero b-value, usually in the order of *b* = 1, 000 s/mm^2^.

We have selected migraine as a case study. Migraine is an attractive pathology for the evaluation of the quality of alternative diffusion metrics, since the differences between patients and controls that have been found using dMRI in the literature are scarce and subtle (Planchuelo-Gómez et al., 2020b). In migraine, differences are usually hard to find in comparison with other disorders such as schizophrenia or Alzheimer's disease, and they require a large number of subjects per group and good quality data. Thus, migraine will allow us to check the capability of different techniques to detect subtle changes.

Migraine is a disabling primary disorder characterized by recurrent episodes of headache, which usually last 4-72 hours and present at least two of the following four characteristics: moderate to severe pain intensity, unilateral location, pulsating quality, and aggravation with physical activity (Third edition of the International Classification of Headache Disorders, ICHD-3). A common distinction when studying migraine is made between episodic migraine (EM), in which patients suffer from headache less than 15 days per month, and chronic migraine (CM), in which patients suffer from headache at least 15 days per month.

A recent study identified statistically significant differences in migraine using advanced diffusion measures calculated with AMURA (Planchuelo-Gómez et al., 2020c). This study identified higher RTOP values in CM patients compared to EM, and lower RTPP values in EM compared to HC.

Given the fact that AMURA-derived measures have shown promising results for the characterization of subtle WM changes in migraine, the main objective of this study was the assessment of the reliability and the robustness of AMURA metrics acquired with a typical acquisition employed in a clinical context. Our purpose is to validate the viability of these metrics for clinical studies even when acquisition protocols are suboptimal for this methodology. Specifically, we will use migraine as a case study and DTI-type acquisitions, where only one shell is acquired at *b* = 1, 000 s/mm^2^.

## 2. Materials and methods

### 2.1. Advanced diffusion measures from single shell acquisitions: AMURA

AMURA was proposed in Aja-Fernández et al. (2020) as a methodology to calculate advanced diffusion metrics from reduced acquisitions compatible with commercial scanners and general clinical routine. It allows the estimation of different diffusion-related scalars using a lower number of samples with a single-shell acquisition scheme. AMURA considers that, if the amount of data is reduced, a restricted diffusion model consistent with single-shell acquisitions must be assumed: the (multi-modal) apparent diffusivity does not depend on the *b*-value, so that a mono-exponential behavior is observed for every spatial direction. According to Basser and Jones (2002), in the mammalian brain, the mono-exponential model is predominant for values of *b* up to 2,000 s/mm^2^ and it can be extended to higher values (up to 3,000 s/mm^2^) if appropriate multi-compartment models of diffusion are employed.

This methodology allows shorter MRI acquisitions and very fast calculation of scalars. Since the mono-exponential model only holds within a limited range around the measured *b*-value, the measures derived this way must be seen as *apparent* values at a given *b*-value, related to the original ones but dependent on the selected shell. The AMURA metrics used in this work are (Aja-Fernández et al., 2020, 2021, 2022):

1. Return-to-origin probability (RTOP), also known as probability of zero displacement, it is related to the probability density of water molecules that minimally diffuse within the diffusion time τ.
2. Return-to-plane probability (RTPP), which is a good indicator of restrictive barriers in the axial orientation.
3. Return-to-axis probability (RTAP), an indicator of restrictive barriers in the radial orientation.
4. Apparent Propagator Anisotropy (APA), an alternative anisotropy metric. It quantifies how much the propagator diverges from the closest isotropic one.
5. Diffusion Anisotropy (DiA), an alternative derivation of APA.
6. Generalized Moments, specifically we will consider the full moments of order 2 (q-space Mean Square Displacement, qMSD) and 1/2 (ϒ^1/2^).

### 2.2. Dataset

#### 2.2.1. Participants

The sample of this study was originally composed of 56 patients with CM, 54 patients with EM and 50 healthy controls (HC) that participated in previous studies (Planchuelo-Gómez et al., 2020a,b). Three patients with EM were discarded due to misregistration errors.

Inclusion criteria included diagnosis of EM or CM following the ICHD-3 (all the available versions), stable clinical situation, and first screening related to migraine just before the recruitment. Exclusion criteria were use of preventive treatments before the MRI acquisition, migraine onset in people older than 50 years, recently developed migraine (less than 1 year), frequent painful conditions, psychiatric and neurological disorders different to migraine, and pregnancy. Further details are available at Planchuelo-Gómez et al. (2020b).

The local Ethics Committee of Hospital Clínico Universitario de Valladolid approved the study (PI: 14-197). Additionally, all participants read and signed a written consent form prior to their participation.

The detailed demographic and clinical features of the three groups are shown in Table 1. No statistically significant differences in age or gender were found between the three groups. Patients with CM showed significantly higher duration of migraine, frequency of headache and migraine attacks and medication overuse, and a lower presence of aura.

**Table 1:** Clinical and demographic characteristics of healthy controls (HC), episodic migraine (EM), and chronic migraine (CM).

	**HC (*n* = 50)**	**EM (*n* = 51)**	**CM (*n* = 56)**	**Statistical test**
Gender, male/female	11/39	7/44	6/50	$\chi_{\left(2,N=157\right)}^{2}$ = 2.74
Age (years)	36.1 ± 13.2	36.6 ± 7.9	38.1 ± 8.7	$\chi_{\left(2\right)}^{2}$ = 2.79, *p* = 0.25^‡^
Duration of migraine history (years)		13.1 ± 10.5	19.6 ± 10.4	U = 932.5, *p* = 0.002^◇^
Time from onset of chronic migraine (months)			24.5 ± 32.9	
Headache frequency (days/month)		3.6 ± 1.9	23.3 ± 6.3	U = 40.0, *p* < 0.001^◇^
Migraine frequency (days/month)		3.6 ± 1.9	13.9 ± 6.9	U = 99.5, *p* < 0.001^◇^
Medication overuse		0 (0%)	42 (75%)	*p* < 0.001^*^
Aura		9 (18%)	1 (2%)	*p* = 0.006^*^

^†^Chi-square test. ^‡^Kruskal-Wallis test. ^◇^Mann-Whitney *U*-test. ^*^Fisher's exact test. Data are expressed as means ± SD.

#### 2.2.2. MRI acquisition

For patients with migraine, the images were acquired at least 24h after the last migraine attack and before 2 weeks after the clinical visit to the headache unit. High resolution 3D T1-weighted followed by DWI were acquired using a Philips Achieva 3T MRI unit (Philips Healthcare, Best, The Netherlands) with a 32-channel head coil.

The acquisition of T1-weighted images was carried out using a Turbo Field Echo sequence with the following parameters: repetition time (TR) = 8.1 ms, echo time (TE) = 3.7 ms, flip angle = 8^*o*^, 256 × 256 matrix size, spatial resolution of 1 × 1 × 1 mm^3^ and 160 sagittal slices covering the whole brain.

The acquisition parameters for DWI were TR = 9,000 ms, TE = 86 ms, flip angle = 90^*o*^, 61 diffusion gradient orientations, one baseline volume, *b*-value = 1,000 s/mm^2^, 128 × 128 matrix size, spatial resolution of 2 × 2 × 2 mm^3^ and 66 axial slices covering the whole brain.

All the images were acquired in the same session with a total acquisition time of 18 min.

### 2.3. Analysis of the data

#### 2.3.1. dMRI preprocessing

Image preprocessing steps consisted of (1) denoising based on the Marchenko-Pastur Principal Component Analysis procedure (Veraart et al., 2016), (2) eddy currents and motion correction, and (3) correction for B1 field inhomogeneity. The MRtrix software (Tournier et al., 2019) was employed to carry out these steps, using the *dwidenoise, dwipreproc*, and *dwibiascorrect* tools (Zhang et al., 2001; Smith et al., 2004; Andersson and Sotiropoulos, 2016; Veraart et al., 2016). Further, a whole brain mask for each subject was obtained with the *dwi2mask* tool (Dhollander et al., 2016).

#### 2.3.2. Diffusion measures estimation

Two groups of diffusion measures were extracted. The former group is composed of three DTI classical metrics: FA, MD, axial diffusivity (AD), and radial diffusivity (RD). We considered only these measurements as they are the ones employed in most previous studies, particularly in the literature migraine, with no studies applying other measurements excluding the one carried out with this sample or the use of kurtosis (Ito et al., 2016).

These measures were estimated at each voxel using the *dtifit* tool from the FSL software (Jenkinson et al., 2012). FA measures the degree of anisotropy in the diffusion of water molecules inside each voxel, which reflects the degree of directionality of water diffusivity. MD is the average magnitude of water molecules diffusion. AD measures the water diffusion in the principal direction of WM fibers. RD describes the perpendicular diffusion of the principal direction (Pelletier et al., 2016).

**Figure 1:**
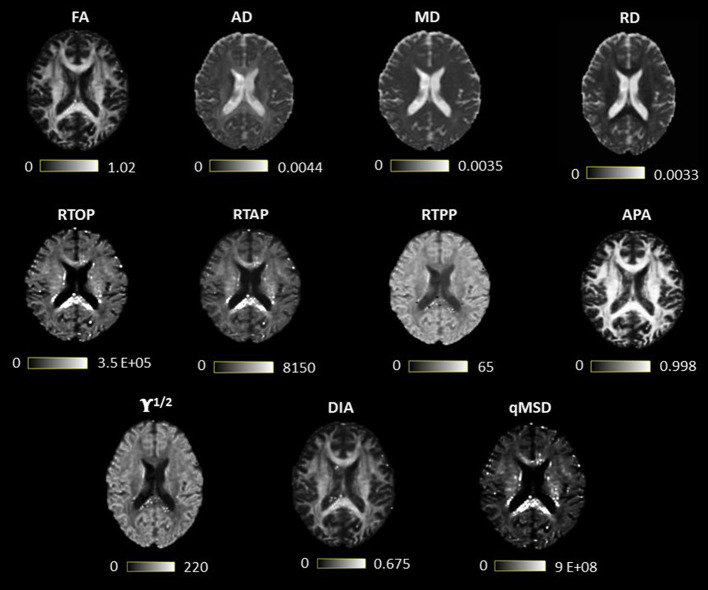
Visual comparison of diffusion tensor imaging (DTI) and measures from apparent measures using reduced acquisitions (AMURA). The first row contains the DTI measures, and the last two, the AMURA metrics.

The latter group includes the seven proposed q-space metrics calculated with AMURA: RTOP, RTAP, RTPP, APA, qMSD, DiA, and ϒ^1/2^. The measures were calculated using dMRI-Lab^1^ and MATLAB 2020a. AMURA measures rely on the expansion of spherical functions at a given shell in the basis of spherical harmonics (SH). Even SH orders up to six were fitted with a Laplace-Beltrami penalty λ = 0.006. A fixed value of τ = 70 ms has been assumed for all the AMURA metrics. A visual comparison of the DTI and AMURA measures is shown in Figure 1.

### 2.4. Experiment with synthetic data

The main hypothesis of this work is that AMURA metrics are able to detect different diffusion properties than DTI in the white matter. In order to quantify this assumption, an illustrative synthetic experiment was carried out. We simulated a simple diffusion model that diverges from the diffusion tensor (DT). The simplest case is a 2-compartment model in which we considered that the main anisotropic diffusion was ruled by a zeppelin-shaped compartment (Alexander, 2008) and there was an isotropic compartment that stands for the free water fraction (Tristán-Vega et al., 2022):


$$S\left(b\right)=f\cdot Z_{p}\left(b,d_{\left|\right|},d_{⊥}\right)+\left(1-f\right)\cdot \exp\left(-bD_{0}\right)$$

where *Z*_*p*_() is the zeppelin compartment, *D*_0_ is the diffusivity of free water at body temperature (nearly 3.0·10^−6^ μm^2^/s), *d*_||_ (μm^2^/s) and *d*_⊥_ (μm^2^/s) are the parallel and perpendicular diffusivities that model the zeppelin and (1−*f*) is the free-water fraction.

For the experiment, different values of *f* were considered, ranging from 0.3 to 1. The value of *d*_||_ was fixed and *d*_⊥_ was changed as a function of *f* for two different cases

1. The FA obtained after estimating the DT from *S*(*b*) is constant.
2. The MD obtained after estimating the DT from *S*(*b*) is constant.

Sixty-one gradient directions and *b* = 1,000 s/mm^2^ were considered. DTI and AMURA metrics were calculated from the synthetic signal.

In comparison with previous studies that assessed EAP-derived measures (Fick R. H. J. et al., 2016; Zucchelli et al., 2016), we employed a simpler model due to the different objective of our study. The previous studies were focused on a detailed characterization of the microstructure with the assessment of the sensitivity of the EAP measures under different conditions with a three-compartment model. The intracellular volume fraction and dispersion were additionally included compared to our experiment for the three-compartment model. In our study, the main objective was the assessment of AMURA measures compared to DTI in the context of clinical studies, i.e., comparison between clinical groups, with a reduced dMRI acquisition. Therefore, this synthetic experiment worked as a proof of concept to appreciate different properties of the AMURA and DTI measures, and not as a detailed analysis of the parameters in relation to microstructural features.

### 2.5. Statistical analysis

#### 2.5.1. ROI analysis and TBSS

To test the capability of AMURA measures at *b* = 1,000 s/mm^2^ to be used in clinical studies, two different statistical analyses were considered: a region-oriented analysis and tract-based spatial statistics (TBSS) assessment. For both approaches, statistical differences between EM, CM, and HC were assessed with two-by-two comparisons. Forty-eight different regions of interest (ROIs) were identified using the Johns Hopkins University ICBM-DTI-81 White Matter Atlas (JHU WM) (Oishi et al., 2008). The first steps of the two assessing methods were common. The FA volumes were non-linearly registered to the Montreal Neurological Institute (MNI) space using the JHU WM template as reference. In the MNI space, the mean FA image for all the subjects was extracted and it was used to generate the white matter skeleton using a minimum FA value of 0.2. For each subject, the FA values were projected to the skeleton. For all the non-FA measures, the same registration used for the FA maps and projection to the skeleton obtained from the FA volumes were carried out.

For the ROI-based analysis, to obtain more robust measures, the average value of the metrics for each subject was obtained using voxels exclusively included in the white matter skeleton within the 2% and 98% percentiles of the corresponding skeleton values. Then we carried out a two-sampled-two-tailed, pooled variance *t*-test between each pair of groups (EM-HC, CM-HC, and EM-CM) for every measure and ROI.

The TBSS approach was conducted to mimic a clinical study following the basic procedure implemented in Planchuelo-Gómez et al. (2020c) and Planchuelo-Gómez et al. (2020b). In this assessment, the statistical comparisons were conducted using the *randomize* tool from FSL (Nichols and Holmes, 2002), which performs a permutation test. Specifically, 5,000 permutations and the threshold-free cluster enhancement (TFCE) procedure were employed (Smith and Nichols, 2009). Briefly, TFCE enhances zones of the voxelwise statistic maps that show spatial contiguity to obtain spatial clusters without using specific values to delimitate different spatial areas with similar values. We considered that an atlas-defined region presented statistically significant differences, after family-wise error correction and TFCE, when the voxels with differences contained a volume greater than 30 mm^3^. Each ROI from the atlas could be part of one or more clusters defined by the TFCE procedure, i.e., TFCE was applied independently from the JHU WM atlas and the voxels for each region were extracted within the defined clusters by TFCE.

The threshold for statistical significance for all the statistical assessments was *p* < 0.05. It is worth noting that the purpose of the ROI-based analysis was not to carry out a complete and accurate clinical study, but to analyze the behavior of each measure separately. Thus, the results in this case were not corrected for multiple comparisons, causing some variations with the results reported in the literature. For the same reason, clinical covariates were not included in all the statistical comparisons.

Further, in relation to the ROI-based analysis, Cohen's D value was calculated over the different ROIs to quantify the effect size of the different DTI and AMURA scalars. In addition, the Cohen's *D*-value was obtained for the full WM to better describe what happened with each measure in the whole brain.

#### 2.5.2. Resampling of diffusion measures

To better understand the discrimination power of each measure, we analyzed their statistical significance in relation to the number of subjects in each group, i.e., the sample size. To that end, a resampling experiment was carried out. The number of subjects of each group (EM, CM, and HC) was progressively decreased from the original number to 10 subjects in each group, reducing five subjects for each iteration. For each iteration, 5,001 different subsamples were randomly obtained following a bootstrapping procedure. For each subsample, the ROI-based approach described in Section 2.5.1, i.e., the uncorrected *t*-tests of the diffusion descriptors from each JHU WM atlas ROI within the WM skeleton, was repeated. Specifically, for the tests with statistically significant differences in the reference comparisons with the whole sample, two-by-two comparisons between HC, EM, and CM groups were carried out. For each ROI, diffusion metric and specific configuration, a ROI was considered to have significant differences if at least the two-by-two comparison in 2,501 out of the 5,001 subsamples showed *p* < 0.05, value established as threshold for statistical significance, as in the whole sample. No kind of statistical correction was used for this experiment considering that our purpose was to study the behavior of the different metrics with the sample size.

#### 2.5.3. Analysis of stability

The coefficient of quartile variation (CQV) was used to measure the stability across groups. The CQV is a measure of homogeneity (Altunkaynak and Gamgam, 2019) and it was used to assess the inter-subject variability, considering the diverse sample sizes from the analysis described in the previous section. The CQV is one of the most robust statistical measures as it depends on the quartiles, being less sensitive to outliers. Its use is as follows:


(1) $$\begin{array}{l} \text{CQV}=\frac{Q_{3}-Q_{1}}{Q_{3}+Q_{1}}\cdot 100 \end{array}$$

where *Q*_1_ and *Q*_3_ are the first and third quartile, respectively.

The CQV is calculated for each group and ROI, considering as figure of merit the median value of all the CQV of the different 5001 subsamples used in this experiment. The 95% confidence interval (95% CI) was set taking the 2.5 and 97.5 percentiles of the whole CQV values for each group of values. This 95% CI was compared between the diverse measures and regions for each sample size.

## 3. Results

### 3.1. Experiment with synthetic data

**Figure 2:**
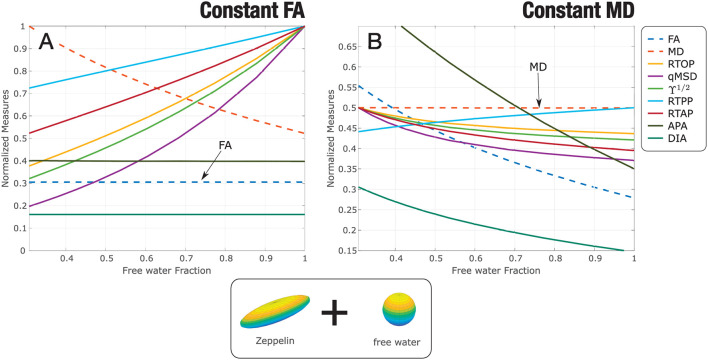
Experiment with synthetic data: a two-compartment model is considered, zeppelin + free water. The parameters of the zeppelin are modified so that the estimated diffusion tensor in every case shows: **(A)** constant FA; **(B)** constant MD. AMURA metrics have been calculated. Measures are normalized for better visualization.

Results for the experiment with synthetic data are gathered in Figure 2: constant FA (Figure 2A) and constant MD (Figure 2B). All measures have been normalized for better visualization and comparison. When FA is set to constant, in this simple scenario, anisotropy-related metrics (PA and DiA) behave similarly. The other AMURA metrics detect the underlying change and grow with *f*, presenting qMSD, ϒ^1/2^ and RTOP similar but higher slopes of opposite sense compared to MD, which decreases with *f*, begin the change of the RTAP almost identical to the one shown by the MD. On the other hand, when MD is set to constant, Figure 2B, all the AMURA measures are able to detect the changes in the signal, and the DiA presented a similar steep rate compared to the FA, and higher steep rate values were appreciated in the case of APA. This example illustrates that, although interpretation of some AMURA measures can be similar to DTI measures, they are not really quantifying the diffusion signal in the same way. The variety of AMURA measures allows not only to detect similar patterns compared to DTI, but also to find complementary results.

### 3.2. ROI based statistical analysis

Eleven different measures were considered for the analysis: four DT-based measures (FA, AD, MD, RD) and seven AMURA-based (RTOP, RTAP, RTPP, qMSD, ϒ^1/2^, APA, and DiA). Table 2 shows a *p*-value scheme for the 48 ROIs considered for each of the measures. Those ROIs that exhibit differences with statistical significance above 95% (*p* < 0.05) are highlighted in green and above 99% (*p* < 0.01) in amber. The size of the effect (Cohen's D) is shown for those ROIs with significant differences (in bold face those values in which *D*>0.5).

**Table 2:**
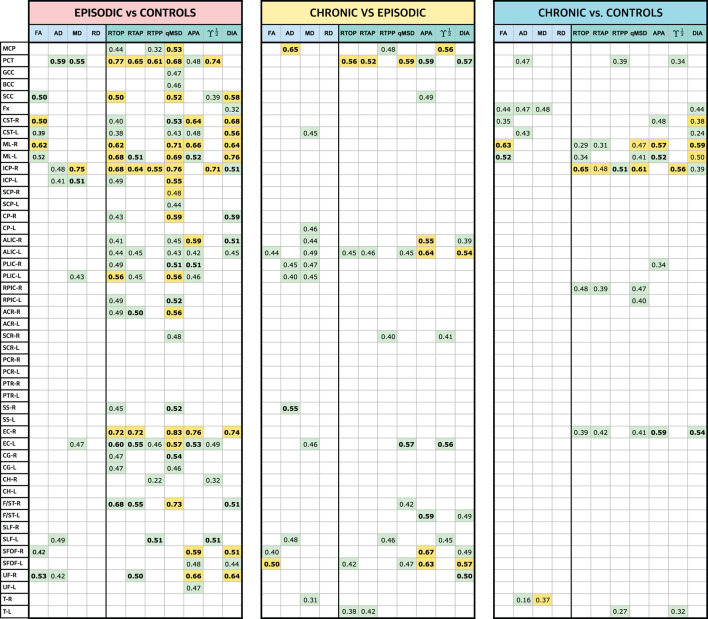
Results of the ROI-based statistical analysis and Cohen's D: EM vs. HC, CM vs. EM, and CM vs. HC.

Two-sample *t*-tests for DTI and AMURA measures and each of the ROIs defined by thee JHU WM atlas. The *p*-values represent the probability that a certain measure has identical means for both groups. ROIs exhibiting differences with statistical significance above 95% (*p* < 0.05) are marked in green and above 99% (*p* < 0.01) in amber. The Cohen's D of those ROIs showing statistical differences is included.

Note that those metrics based on the DT showed a limited amount of differences with only three ROIs with statistically significant differences above 99% for EM vs. CM, two for CM vs. EM and two for CM vs. HC. In the EM vs. HC comparisons, the highest differences between AMURA and DTI metrics, with a greater number of statistically significant results for AMURA, were found: even in those cases in which the DT found differences, like the pontine crossing tract (PCT), the equivalent AMURA metrics showed a smaller p-value and higher effect sizes. RTOP, qMSD, and DiA were the metrics providing a higher number of statistically significant differences with the higher significance (see amber ROIs) and the greater effect size.

Regarding the other two sets of comparisons (CM-EM and CM-HC), AMURA metrics showed no clear higher number of differences compared to DTI metrics. In fact, AD and MD were able to detect more differences in the comparisons between CM and EM, coherently with previous studies (Planchuelo-Gómez et al., 2020b). This case suggests the complementary nature of DTI and AMURA. As shown in the preliminary example, both methods are quantifying different microstructure effects. Thus, AMURA seems more sensitive to changes between EM and controls, while DTI seems more sensitive to changes between the two types of migraine.

It is important to note that in all three comparisons, RD did not find any significant differences in any ROI, which is consistent with the findings reported in Planchuelo-Gómez et al. (2020b). Therefore, to streamline the presentation of data in the figures and tables that follow, RD will be omitted in the following experiments.

**Figure 3:**
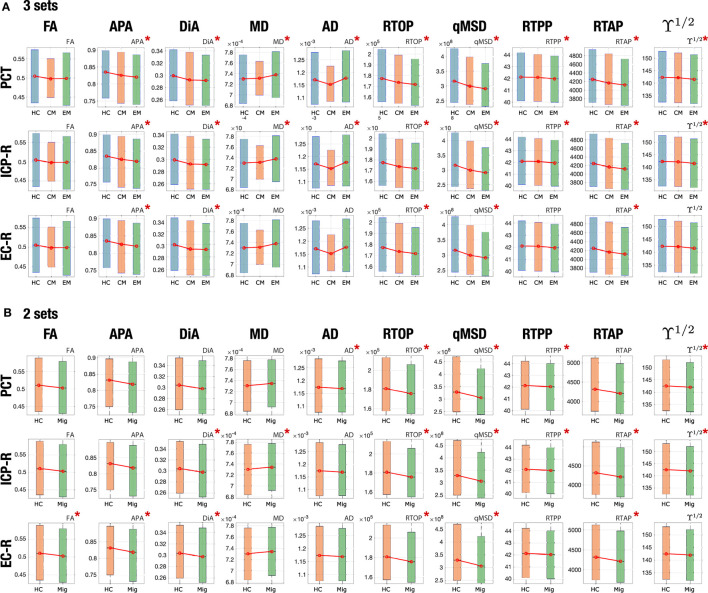
Boxplots of the distribution of different measures for EM, CM, and HC for three specific regions: PCT, ICP-R, and EC-R. The star marks those regions with statistically significant differences in the ROI analysis. **(A)** 3 sets (HC, CM, EM). **(B)** 2 sets (HC and migraine).

To better understand the behavior of both sets of measures, let us deeply analyze three specific regions. We selected the PCT, right inferior cerebellar peduncle (ICP-R) and the right external capsule (EC-R) for being the ones with the highest number of differences and the greatest effect sizes in Table 2. For each ROI, a box plot of the three groups is shown for each measure in Figure 3A. The boxes mark the median and 25 and 75 percentiles of the values of the different measures over the skeleton of the FA for all the subjects in each group. For better visualization, the median of each group is marked in red. The box plots are repeated in Figure 3B merging EM and CM in a single group that includes all migraine patients.

In the PCT, regarding DTI, the statistical analysis found differences between EM and HC for MD and AD, and between CM and HC for AD, but no differences were found between both migraine groups. In Figure 3A, we can see that, actually, MD showed a higher median value of EM and CM when compared with HC. These differences were kept in Figure 3B when considering the joint migraine group. On the other hand, AMURA showed significant differences between EM-HC and CM-EM. Only RTPP (a metric related to AD) and ϒ^1/2^ Regarding the other two sets of comparisons (CM-EM and CM-HC), AMURA metrics showed no clear higher number of differences compared to DTI metrics. In fact, AD and MD were able to detect more differences in the comparisons between CM and EM. This would mean that AMURA better discriminates EM in this ROI. According to Figure 3A, that is precisely what is happening. See, for instance, RTOP and qMSD. In both cases, there is almost no difference between HC and CM, while EM shows smaller median and a reduced variance. On the other hand, RTPP behaves more similarly to AD: both migraine groups were similar but differ from the control-group.

For the ICP-R, according to Table 2, MD and AD differences were found for the EM-HC case, AMURA found differences for EM-HC and CM-HC and no differences were found for CM-EM. If we check Figure 3A we can see that both migraine groups presented similar values in this ROI. Statistically significant differences were found between CM and HC, presenting the RTOP, qMSD, and RTAP lower values in CM.

A similar effect can be observed in the EC-R, where no differences were found for DTI parameters, but for AMURA in the comparisons between HC and the two migraine groups. If we see Figure 3A, we can observe that AMURA metrics (RTOP and qMSD, for instance) discriminated CM and HC better than MD and AD. While in the MD and AD cases there is a reduction in the variance of the CM group, the change in the median is smaller, compared to CM and EM. If we pay attention to Figure 3B, we can see migraine and HC showed similar AD and MD values, while differences could be appreciated with RTOP, qMSD, and RTAP.

### 3.3. Effect size

**Figure 4:**
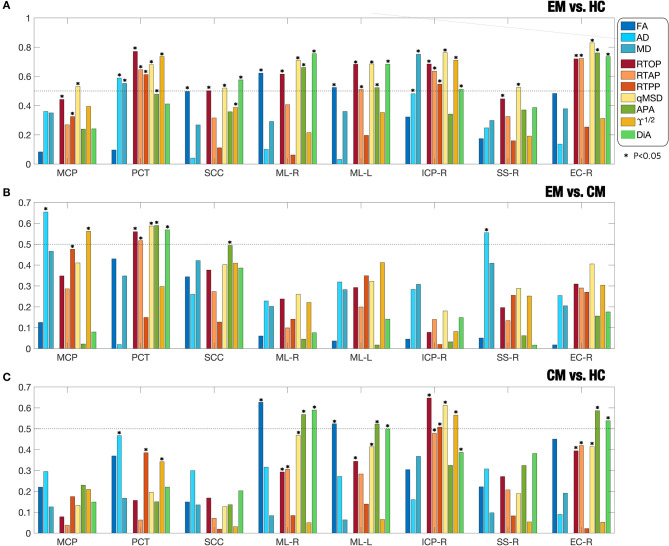
Absolute value of effect sizes (absolute Cohen's d) for associations between **(A)** EM and HC; **(B)** EM and CM; **(C)** CM and HC. Different DTI and AMURA metrics are considered for eight selected ROIs (MCP, PCT, SCC, ML-R, ML-L, ICP-R, SS-R, EC-R) according to the JHU WM Atlas. The star marks those regions with statistically significant differences in the ROI analysis.

In Table 2, the values of the Cohen's D were shown for those ROIs with significant differences. Figure 4 shows the absolute value of Cohen's D for eight selected ROIs (those with the largest number of differences) and for the three group comparisons.

The comparison between EM and HC, the AMURA metrics showed the largest effect sizes as measured by larger Cohen's *D*-values. Specifically, qMSD, RTOP, and DiA were consistently getting values over 0.5 (the threshold for medium effect) and, in some cases, near 0.8. In the right external capsule (EC-R), for instance, most AMURA metrics showed a moderate-large effect size while DTI metrics did not get to 0.5. Even in those regions where DTI values showed statistical differences and a moderate effect (PCT, ML-R), AMURA outperformed them. There is only one case, the MD in the ICP-R, where a DTI metric showed a moderate effect size. However, if we check Table 2, we can see that the effect size for MD is 0.75, but this value was slightly lower than the value for the qMSD (0.75 vs. 0.76).

Regarding the comparison between CM and EM (Figure 4B), most measures showed low effect sizes, both for DTI and AMURA. The middle cerebral peduncle (MCP) for the AD and the right sagittal stratum (SSR) showed Cohen's *D*-values over 0.5 for the AD, while AMURA only achieved medium effects in the pontine crossing tract (PCT).

Finally, in the comparison between CM and HC (Figure 4C), the right external capsule (EC-R), the right medial lemniscus (ML-R) and the left medial lemniscus (ML-L), the APA and the DiA reached absolute values of Cohen's D higher than 0.5, showing at the same time significant differences. FA also showed moderate effect in ML-R and ML-L, while RTOP, qMSD, and ϒ^1/2^ showed values over 0.5 in the right inferior cerebellar peduncle (ICP-R).

**Figure 5:**
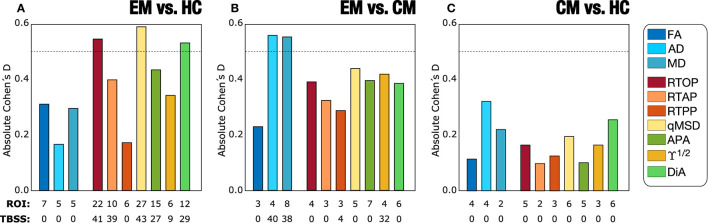
Absolute value of Cohen's *D*-values for the three group comparisons in the Full WM: **(A)** Episodic Migraine (EM) vs. Healthy Controls (HC); **(B)** EM vs. Chronic Migraine (CM); **(C)** CM vs. HC. DTI and AMURA measures are depicted. For each measure, the total number of ROIs that presented statistically significant differences obtained with the ROI and TBSS statistical approaches are also noted.

It is also interesting to analyze the behavior of each measure over the whole WM. Figure 5 shows the absolute Cohen's D in the whole WM for each measure. The biggest effect sizes were obtained for the comparison between EM vs. HC for AMURA. Coherently, this comparison also produced the highest number of ROIs with significant differences. The qMSD or the RTOP measures reached absolute Cohen's *D*-values close to 0.6, and, respectively, 27 and 22 ROIs with significant differences for the ROI analysis, 43 and 41 in TBSS. On the other hand, the comparison between CM and HC presented the lowest Cohen's *D*-values, none of them reaching 0.5. Regarding the comparison between CM and EM, the AD, MD were the measures with greatest Cohen's *D*-values, over 0.5.

### 3.4. Change of the sample size

**Figure 6:**
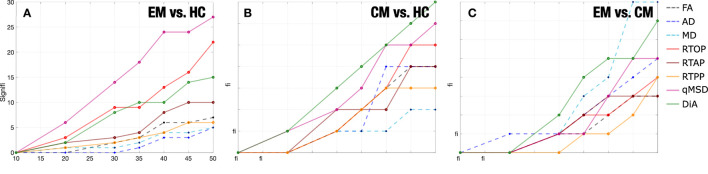
Number of ROIs with statistically significant differences by the resampling of diffusion measures reducing the number of subjects per group (sample size). **(A)** Episodic Migraine (EM) vs. Healthy Controls (HC). **(B)** Chronic Migraine (CM) vs. HC. **(C)** CM vs. EM. No statistical correction was considered. For each case the median of 5,001 permutations considered.

Figure 6 shows the effects of changing the sample size for different DTI and AMURA-based measures for the three comparisons considered. We have selected 8 out of 11 metrics for better visualization of the graphics. Among the DTI measures, results with MD showed a relatively high number of ROIs with statistically significant differences using bigger samples sizes, especially for the EM-CM comparison, as can be seen in Figure 6C. However, even in that case, the number of significant ROIs drastically decreased for a group sample size of 40. In addition, few ROIs with statistically significant differences were found for the rest of DTI measures and for the other two group comparisons, in any sample size, which made the assessment of the relationship between DTI measures and sample size unfeasible.

Results showed a stable behavior of AMURA measures in relation to the sample size, which can be understood as a linear dependence between the group sample size and the number of statistically significant ROIs. In Figure 6A, this behavior can be better understood and interpreted in measures such as qMSD, which was the most robust one in the comparison between EM and HC. Furthermore, RTOP, qMSD, and DiA also showed a robust behavior in the CM vs HC comparison. Notice that AMURA measures reached the lack of statistical significance ROIs for a group sample size of 10. However, when reducing the sample size to half (*N* = 25), most AMURA metrics still were able to find differences between groups, while only a few differences remained for the DTI case.

**Figure 7:**
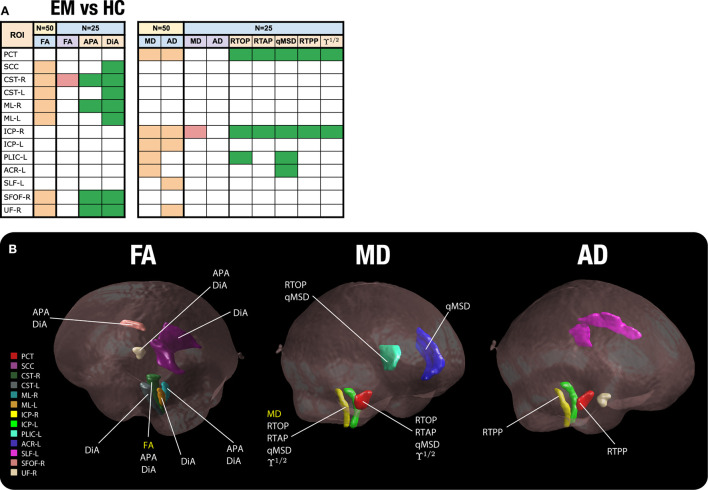
Significant ROIs found for a reduced sample size (*N* = 25). **(A)** Table of ROIs found at *N* = 25 compared to the original sample size (EM vs. HC). In amber, the ROIS with differences for DTI at the original sample size; in red, those ROIS with differences for DTI for a reduced sample size (*N* = 25); in green, those ROIS with differences for AMURA for a reduced sample size (*N* = 25). **(B)** The 13 ROIs detected by DTI at the original sample size are shown in the white matter. For each ROI, we have added the label of those metrics that show significant differences for a sample size of *N* = 25.

In order to better understand this effect, we now analyze the behavior of the measures in selected ROIS. We have chosen, according to results in Table 2, those 13 regions in which DTI measures showed differences with the original sample size for EM vs. HC (see Figure 7A). For those 13 ROIs, in 7 of them FA showed significant differences for *N* = 50, 5 for MD and 5 for AD (see ROIs marked in amber). Then, we look at the results for those specific ROIs for a reduced sample size of *N* = 25. Note that, in that case, when the number of subjects is reduced to half, the FA was only able to detect one ROI (out of 7), MD only one (out of 5), and AD none (ROIs marked in red). When we look to the AMURA metrics, we see that they were able to still keep most of those differences even for a reduced sample size (see ROIs marked in green): DiA and APA, anisotropy measures similar to the FA, were able to, respectively find 4 and 7 out of the original 7 FA ROIs. RTAP, and ϒ^1/2^ succeed in finding 2 of the 5 MD ROIs, while RTOP finds 3 and qMSD 4 of them. In addition, with RTPP, 2 out of the 5 AD ROIs were identified for the reduced sample size. All in all, for this comparison, AMURA outperformed DTI in keeping the differences even for a smaller sample size.

As an illustration, in Figure 7B, the 13 considered ROIS are depicted. For each ROI, the metrics that showed significant differences for a sample size of *N* = 25 are displayed.

### 3.5. TBSS (original sample)

As we have previously stated, the ROI analysis carried out in the previous sections could be an illustrative example of the performance of the different metrics and it gives a valuable insight on the relation among them. However, since no statistical correction was considered, the results could not be acceptable for clinical studies. Thus, in order to mimic an actual clinical study, we have now repeated the analysis using TBSS for the three comparisons.

Using the DTI measures (FA, MD, AD, and RD), statistically significant differences between CM and EM patients were observed for two parameters. Patients with CM showed lower AD and MD values than EM in 40 and 38 out of 48 regions from the JHU-WM Atlas, respectively. No statistically significant differences were found using DTI measures between EM and HC or between CM and HC.

For the AMURA metrics, the comparison between patients with EM and HC showed the highest number of parameters with statistically significant differences. Significant lower RTOP, RTAP, qMSD, APA, DiA, and ϒ^1/2^ values in EM compared to HC were found in 41, 39, 43, 27, 29, and 9 ROIs out of 48, respectively. Concerning the comparison between both groups of patients, higher values in CM compared to EM were identified for the RTPP and ϒ^1/2^ in 4 and 32 regions, respectively.

**Figure 8:**
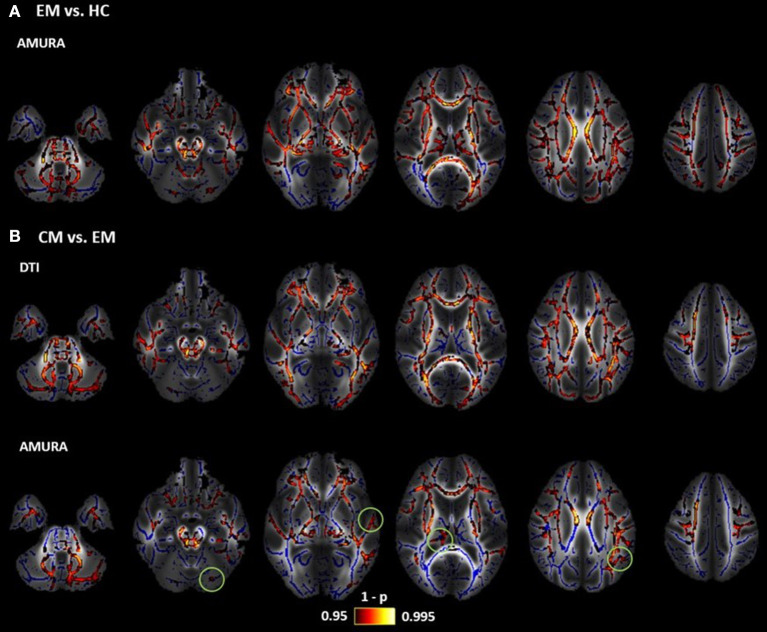
Results of TBSS analysis: statistically significant clusters of voxels distinguishing between DTI and AMURA approaches. Mean FA image at the background, FA skeleton colored in blue and significant ROIs colored in red-yellow. **(A)** Episodic Migraine (EM) vs. Healthy Controls (HC): merged AMURA measures (RTOP, RTAP, APA, qMSD, ϒ^1/2^, and DiA). **(B)** CM vs. EM: merged DTI (AD and MD) and AMURA (RTPP and ϒ^1/2^) measures. DTI measures do not detect any significant ROI either in EM vs. HC nor CM vs. HC. Green circles showed the areas where AMURA measures showed differences in group comparisons where the DTI ones did not.

**Figure 9:**
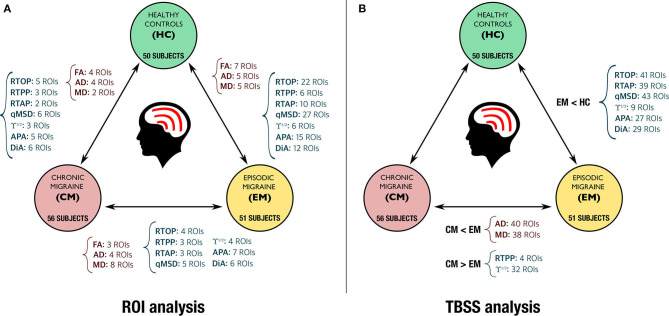
Summary of the statistically significant differences found with DTI (in red) and AMURA measures (in dark blue) for the comparison of the three groups. **(A)** ROI based analysis (no statistical correction). **(B)** TBSS analysis (with family-wise error correction).

Figure 8 shows the TBSS results including all the ROIs that presented statistically significant differences together with the FA skeleton. On the one hand, for EM vs. HC and CM vs. HC comparisons, all the AMURA measures which showed significant differences are merged and depicted in the figure, that is, RTOP, RTAP, APA, qMSD, ϒ^1/2^ and only DiA for EM vs. HC. On the other hand, DTI and AMURA measures can be distinguished in the last CM vs. EM comparison. For DTI, the merged measures depicted are AD and MD, while for AMURA are RTPP and ϒ^1/2^. As it can be seen, AMURA measures showed differences in group comparisons where the DTI ones did not, as shown in the green circles. A summary with the previous TBSS results regarding the number of ROIs and the group comparisons can be found in Figure 9.

### 3.6. Analysis of stability

**Figure 10:**
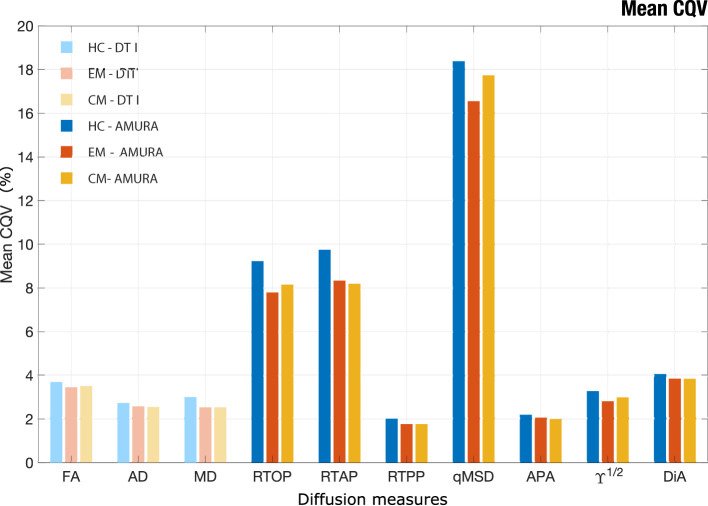
Mean CQV for each group of study considering the 48 ROIs of JHU-WM atlas. Healthy Controls (HC), Episodic Migraine (EM), and Chronic Migraine (CM). DTI and AMURA measures are shown. The measures with the higher stability have lower CQV.

Figure 10 depicts the average values of CQV for all the DTI and AMURA-based diffusion measures. The measures with the highest stability (lowest CQV) were the RTPP and the APA, with an approximate average CQV of 2% considering all the regions. Other measures with relatively high stability were the three DTI measures (FA, MD, and AD), ϒ^1/2^ and DIA, with CQV average values between 2% and 5%. The remaining DTI and AMURA descriptors (RD, RTAP, and RTOP), presented a moderate-high stability, with CQV average values between 5% and 10%. The descriptor with the lowest stability was the qMSD, with CQV average values between 15% and 20%.

Regarding the comparisons between the three groups of interest, after reducing the group sample size to 45 subjects, the assessment of the CQV 95% CI showed that the HC presented a general higher variability than patients with EM and CM. The parameters with a higher number of regions with statistically significant differences between HC and migraine patients according to the 95% CI were the three AMURA measures (RTOP, RTPP, and RTAP) and the MD, with 14–22 regions presenting differences. Additionally, in the comparison between HC and CM, the CQV of APA or ϒ^1/2^ were significantly higher in HC than CM in 13 regions. The number of regions with CQV differences between CM and EM was lower compared to the comparison between HC and the patient groups. FA and MD were the descriptors with a higher number of regions (nine) that showed higher variance in EM compared to CM, and MD was also the parameter with more regions (eight) with significantly higher variance in CM.

## 4. Discussion and conclusions

In this study, we assessed the viability of advanced diffusion descriptors obtained with a novel approach, AMURA, in comparison with traditional DTI parameters. To this end, their capability to discriminate difference between clinical groups of interest was compared, together with the stability of these results for reduced sample sizes. Using synthetic and real data with a single-shell and low b-value, we observed that AMURA is sensitive to changes of parameters associated with the dMRI signal, showing a higher capability of discrimination between clinical groups, even for decreased sample sizes. Specifically, with AMURA we detected a larger number of ROIs with statistically significant differences between groups, or results complementary to those identified with DTI, presenting higher effect size but lower stability than DTI metrics.

Advanced diffusion descriptors such as RTOP, RTAP and APA have shown to be useful for the analysis of the WM of the brain (Aja-Fernández et al., 2020; Planchuelo-Gómez et al., 2020b,c). However, their conventional calculation requires acquisition protocols including several *b*-values, a high number of diffusion gradient directions and very long processing times. This makes them unfeasible for their use in clinical practice or in many commercial MRI scanners. Besides, the use of these metrics in retrospective studies is usually impossible since the acquisition protocols do not allow for it.

AMURA was proposed to allow the estimation of apparent versions of these advanced diffusion measures from reduced acquisitions (Aja-Fernández et al., 2020, 2021, 2022). It provides a fast and straightforward method to compute them from a single shell and very short processing times. Metrics calculated with AMURA have shown a high correlation with measures calculated using a multishell approach, such as MAP-MRI (Özarslan et al., 2013), MAPL (Fick R. H. et al., 2016), or MiSFIT (Tristán-Vega and Aja-Fernández, 2021), for high *b*-values (at least 2,000 s/mm^2^). For lower values, these measures show a weaker correlation since the underlying features measured are better visible at higher *b*-values (Aja-Fernández et al., 2020, 2022). However, we hypothesized that AMURA metrics can still provide useful information at lower *b*-values, complementary to that obtained from DTI-based measures. This paper focuses precisely on that hypothesis and tries to elucidate whether AMURA-based measures obtained from standard DTI-type acquisitions are useful in group studies.

To that end, we have resorted to migraine as our target pathology, because of several reasons. First, diffusion MRI studies in the literature show that differences between patients and HC, or between different groups of patients (EM vs. CM) are subtle, as studies using small sample sizes have often reported no differences and even contradictory findings have been published (Chong and Schwedt, 2015; Messina et al., 2015; Neeb et al., 2015; Gomez-Beldarrain et al., 2016; Shibata et al., 2018; Coppola et al., 2020; Planchuelo-Gómez et al., 2020a).

To study the viability of AMURA-based measures, two different statistical analysis were carried out, including a ROI-based analysis and conventional TBSS, together with the assessment of the behavior of the diverse measures from reduces sample sizes and of the stability. We show that AMURA measures obtained from DTI-type acquisitions were able to successfully find statistically significant differences between the three groups under study (HC, EM, and CM), including differences that were not detected using DTI-based measures. Although AMURA showed additional differences between groups in a preliminary previous study (Planchuelo-Gómez et al., 2020c), the magnitude of the additional differences, particularly those between EM and HC, was unexpected.

With a single-shell and low *b*-value acquisition, AMURA shows itself as a method complementary to DTI, as reflected by the results from the TBSS analysis (Figure 9B). On the one hand, DTI-based AD and MD showed a good performance for the comparison between EM and CM, with a great number of ROIs with statistically significant differences, while AMURA-based measures detected equivalent but a lower number of differences. On the other hand, in the comparison between EM and HC, differences were only found using AMURA-based measures, and in a relatively large number of ROIs. The reason of these differences may be that both techniques represent changes associated with diverse pathophysiological mechanisms, as shown in the example with synthetic data, where only AMURA was able to identify changes of the free water fraction. Further studies on disorders with better characterized pathophysiology than migraine must be carried out to understand the different sensitivity to varied biological processes of DTI and AMURA.

Regarding the behavior of the DTI and AMURA measures in the synthetic experiment for diverse free water fractions, some AMURA parameters showed higher sensitivity to the free water changes. For constant FA, qMSD, ϒ^1/2^, and RTOP presented higher changes for small changes of the free water fraction than the MD, while DiA and APA remained constant. For constant MD, DiA and FA showed similar changes and the APA showed higher changes than the FA, without constant values of any AMURA parameter. These results suggest that AMURA can better determine differences caused by changes of free water fraction in comparison with DTI, as some parameters presented higher sensitivity. Therefore, the consequence would be that AMURA measures may be able to find subtler differences between clinical groups compared to DTI, in line with previously reported results in migraine (Planchuelo-Gómez et al., 2020c).

The complementary nature of DTI- and AMURA-based measures is confirmed by the ROI-based analysis (Table 2 and Figure 9A). In the comparison between CM and EM, for instance, the MD was the metric that detected a higher number of regions with statistically significant differences, but there were some ROIs with differences exclusively identified by one or more AMURA-based measures (e.g., the PCT). In the same way, there were ROIs with differences exclusively found with the MD or AD (e.g., the SS-R), and ROIs with differences identified by both DTI- and AMURA-based measures (e.g., the EC-L).

If we focus on those regions selected in Figure 3 (PCT, ICP-R, and EC-R) we can better understand what is happening with the behavior of the distribution of the different metrics inside the selected ROIs. First, let us focus on the anisotropy measures, FA, APA, and DiA. According to Table 2, there are no differences between groups for the FA for any of the three ROIs. On the other hand, APA and DiA reflected differences for EM vs. HC (for the three ROIs), for EM vs. CM (in the PCT) and CM vs. HC (both in EC-R). These results are confirmed by the boxplots in Figure 3, where AMURA-anisotropy measures were able to better separate the three groups. It is of interest to note that when migraine is considered as a single set, results are more similar for the three metrics, confirming that anisotropy differences between controls are migraine were really present. Regarding the other metrics, AD and MD were able to find significant differences in most cases for the three ROIs, according to Figure 3. However, AMURA metrics always find the same differences but with a greater size effect. As an example of this, we can focus on the PCT for EM vs. HC, where we can see that all the metrics succeed in finding differences but with different effect sizes.

All in all, from the results in Table 2 and Figure 3, we can see that the behavior of AMURA and DTI is similar, although MD and AD showed a lower variance for the CM group. The separation between the groups follows very similar trends within the three ROIs considered. However, AMURA manages to better find these existing differences, and with a larger effect size.

The sensitivity of AMURA-based measures was analyzed by further comparing the effect size found in the different comparisons between groups. A classical method to determine the magnitude of the differences between groups is Cohen's D, which considers the variability of the sample in relation to the average value. As illustrated in Figures 4, 5, DTI-based and AMURA-based measures showed comparable effect sizes for the EM-CM and CM-HC comparisons. In the first case, DTI-based AD and MD reached medium effect sizes (0.5; for the whole WM), while Cohen's D for FA barely exceeded small effect size threshold (0.2). For this last comparison, Cohen's D for AMURA-based measures varied between the small and the medium effect thresholds. For the comparison between CM and EM, however, Cohen's *D*-values were notably lower for all measures, barely reaching 0.3 for DTI-based AD. Finally, regarding the comparison between EM and HC, while DTI-based FA and MD reached Cohen's *D*-values around 0.3, AMURA-based RTOP, qMSD, and DIA reached values over 0.5. These differences in effect sizes among different measures and different group comparisons offer a good explanation for the results shown in Table 2 and Figure 9.

Whereas it may be tempting to think about EM and CM as different degrees of the same pathological process, recent results (Coppola et al., 2020; Planchuelo-Gómez et al., 2020a) support the hypothesis of EM and CM being different entities at the microstructural level, each accompanied by different changes in the WM. Following this hypothesis, DTI-based measures seem well-fitted to detect WM changes in CM, while AMURA-based methods perform remarkably well for the changes that occur in EM. Although the interpretation of changes in DTI or AMURA-based measures is not straightforward, results suggest that WM changes in EM with respect to HC (specifically, lower RTOP and RTAP) might be related to changes in the transverse diffusivity, while changes in CM with respect to EM (such as higher RTPP and lower AD) might be more related to changes in the diffusivity in the axonal or main direction. As previously stated, the complementary use of DTI and AMURA may be useful to detect changes of different nature using data obtained with a low *b*-value and single-shell acquisition. The specific pathophysiological mechanisms related to changes of diverse essence in AMURA must be assessed in future studies.

Considering the difficulty to obtain large sample sizes in group studies, it is important to assess the behavior of the diverse diffusion measures when the number of subjects per group is reduced. As depicted in Figure 6, both DTI-based and AMURA-based measures shared the expected trend, meaning that the number of ROIs with statistically significant differences decreases as the sample size is reduced. However, as shown in the experiment in Figure 7, when the number of samples is reduced to half, DTI metrics were no longer able to detect the differences between groups in most ROIs, whereas AMURA could. From the 13 ROIs considered in the experiment, DTI lost 11 of them when reducing the sample size, while AMURA only lost 2 of them. This effect favors the usage of AMURA metrics in studies with a small sample size.

The assessment of the stability provides another interesting perspective for the evaluation and comparison between different diffusion measures. The diffusion measures that showed higher stability (lower CQV) were AMURA-based APA and RTPP, and the DTI-based measures, while AMURA-based qMSD seems to present low stability. This high variability was expected, since qMSD is a quadratic measure, so it must show a greater range of variability. Interestingly, it presented a relatively high number of regions with statistically significant differences in the comparisons of both migraine groups against controls for diverse sample sizes despite their low stability. Therefore, the results of this study suggest that qMSD is able to characterize specific microstructural properties that are particularly difficult to find with other parameters. Moreover, as it has been suggested previously in this section, differences between both groups of patients with migraine and controls may be qualitatively distinct compared to the differences between CM and EM. Furthermore, qMSD is especially sensitive to short diffusion time scales (Ning et al., 2015).

It is important to note that the AMURA-based measures employed in this paper must be considered as apparent values at a given *b*-value, and their interpretation in terms of the microstructure properties may be different from that of the original EAP-based diffusion measures. Although the relationship between AMURA-based measures and their original counterparts deserves further study, in this paper we deliberately chose not to pursue this comparison to focus on the viability of AMURA-based measures to complement DTI in scenarios where EAP-based measures cannot be obtained.

This study presents limitations that must be pointed out. First, the pathophysiological interpretation of the different trends of the AMURA-based measures is not totally clear, so a description of the microstructural properties according to the values of each measure cannot be provided. As mentioned previously, the apparent nature of AMURA-based measures and their complex relationship with the original EAP-based measures prevent the direct adoption of interpretations from those EAP-based measures. Microstructural studies like those conducted for DTI-based measures (Alexander et al., 2007; Winklewski et al., 2018) are needed to fully understand the results obtained with AMURA.

Furthermore, the results obtained in this study cannot be directly translated to other pathologies affecting the WM of the brain. Even though AMURA can be expected to be a useful information to detect differences in group studies targeting other diseases, further research is needed to confirm that.

In conclusion, this study showed that the new AMURA-based measures can be easily integrated in group studies using single-shell dMRI acquisition protocols, and they can reveal WM changes that may remain hidden with traditional DT-based measures. The wide variety of AMURA, a fast and relatively simple approach, provides measures that allow to extract values that are able to find differences between groups for restricted sample sizes and dMRI acquisition protocols.

## Data availability statement

The datasets used for this study belong to Hospital Clínico Universitario (Valladolid) and cannot be made public. Researchers may request access to them by contacting the corresponding author. Requests to access these datasets should be directed to sanaja@tel.uva.es.

## Ethics statement

The studies involving human participants were reviewed and approved by Comité Ético, Hospital Clínico Universitario de Valladolid. The patients/participants provided their written informed consent to participate in this study.

## Author contributions

CM-M: validation, formal analysis, investigation, writing—original draft, and writing—review and editing. ÁP-G: methodology, validation, formal analysis, investigation, data curation, writing—original draft, and writing—review and editing. ÁG and DG-A: methodology and validation. AT-V: methodology, validation, formal analysis, supervision, project administration, and funding acquisition. RL-G: methodology, validation, formal analysis, investigation, writing—original draft, supervision, project administration, and funding acquisition. SA-F: conceptualization, methodology, software, validation, formal analysis, investigation, writing—original draft, writing—review and editing, supervision, project administration, and funding acquisition. All authors contributed to the article and approved the submitted version.

## Footnotes

^1^Available at www.lpi.tel.uva.es/dmrilab.
