# Erratum: Viability of AMURA biomarkers from single-shell diffusion MRI in clinical studies

**DOI:** 10.3389/fnins.2023.1228337

**Published:** 2023-06-09

**Authors:** Frontiers Production Office

**Affiliations:** Frontiers Media SA, Lausanne, Switzerland

**Keywords:** alternative metrics, AMURA, brain, diffusion magnetic resonance imaging, DTI, migraine

Due to a production error, there was a mistake in Figure 4 and Figure 9 as published. Figure 4 had a formatting issue and part of Figure 9B was missing. The corrected Figure 4 and Figure 9 appear below.

**Figure 4:**
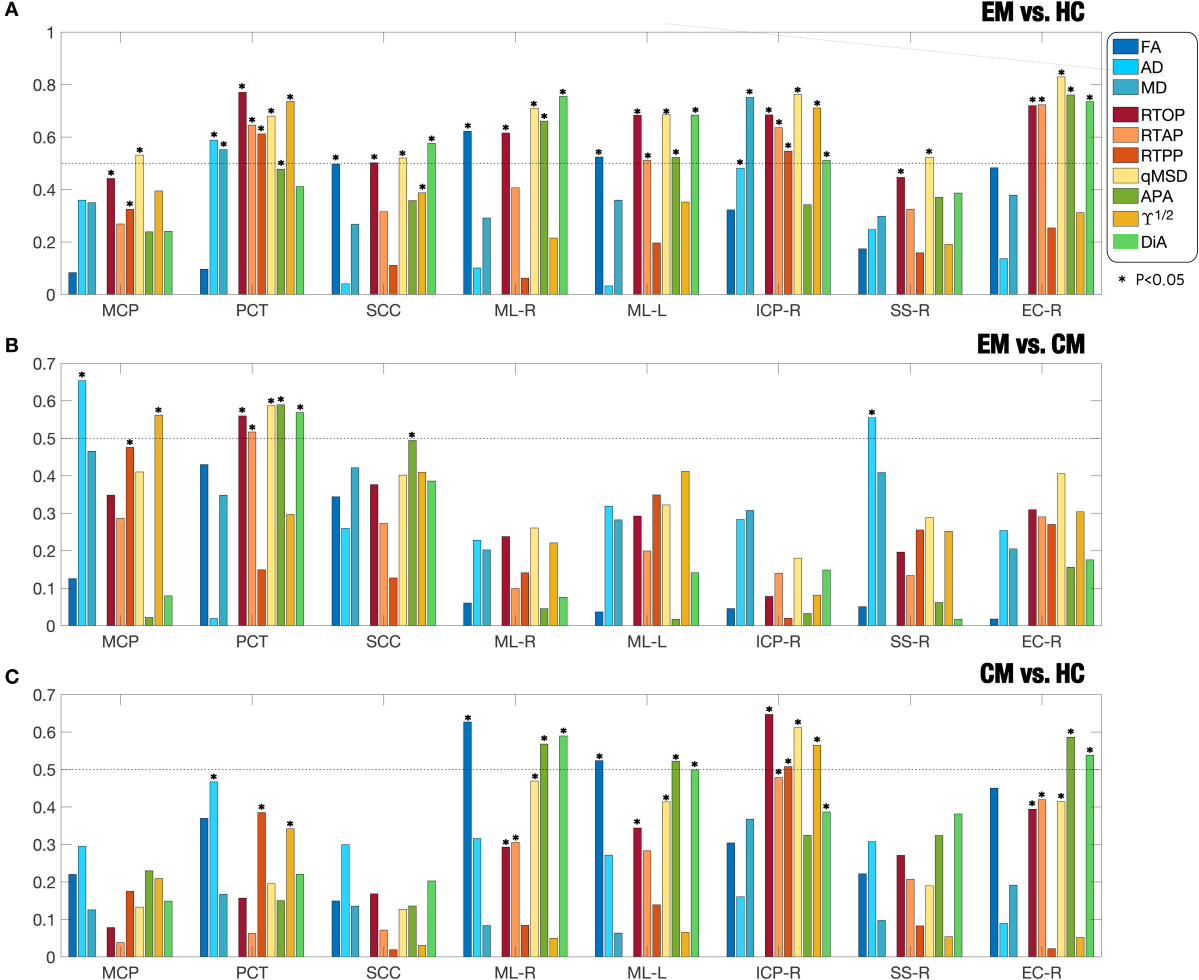


**Figure 9:**
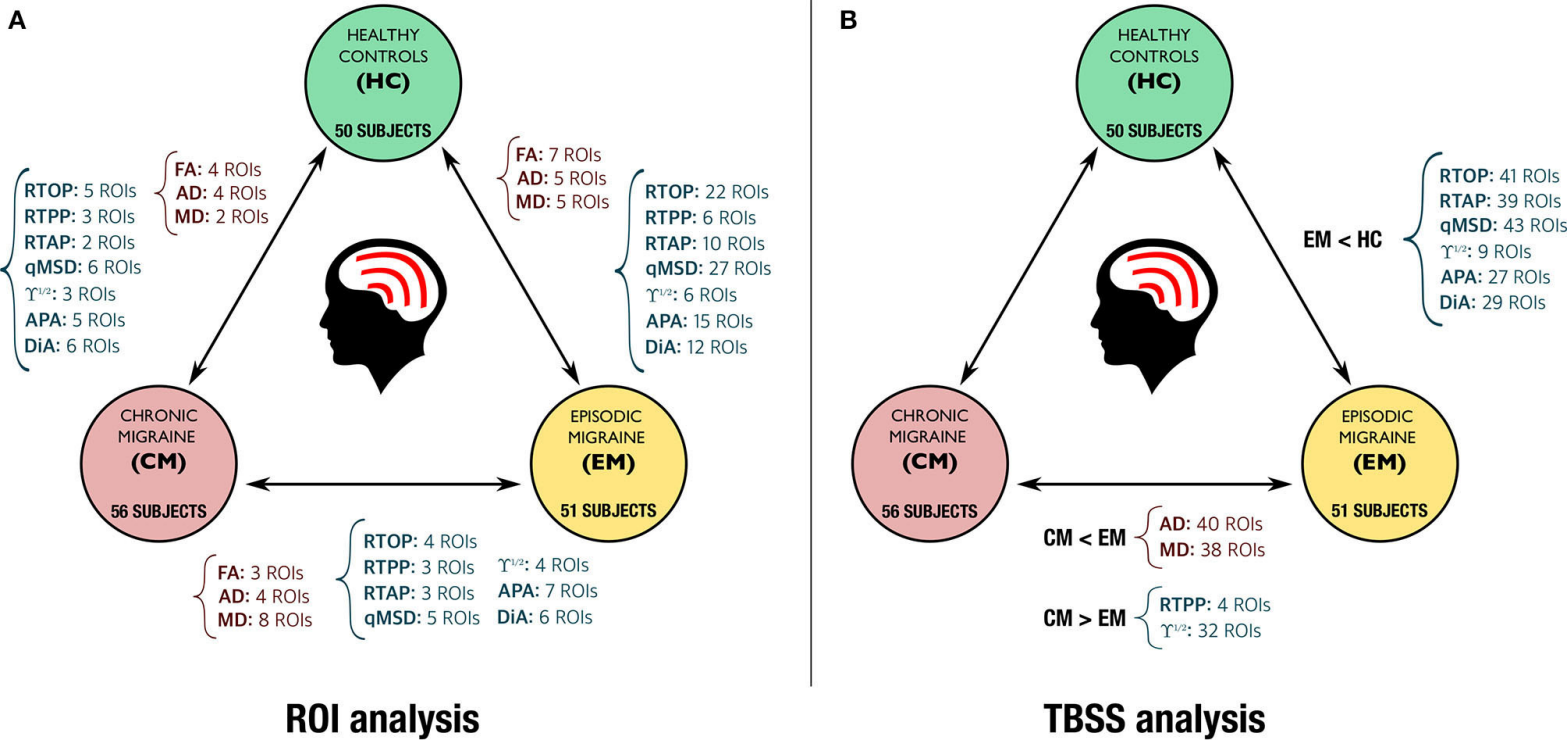


The publisher apologizes for this mistake. The original article has been updated.

