# Supplementary material for: Viability of AMURA biomarkers from single-shell diffusion MRI in clinical studies
Source: Front Neurosci. 2023 May 10;17:1106350. doi: 10.3389/fnins.2023.1106350 (PMC10208402; doi:10.3389/fnins.2023.1106350)
Supplement: Supplementary file 1 [file Data_Sheet_1.PDF]

SUPPLEMENTARY MATTERIAL

Viability of AMURA biomarkers from single-shell diffusion MRI in Clinical Studies

CarmenMartín-Martín[1], Álvaro Planchuelo-Gómez[1,4], Ángel L. Guerrero[2,3], David García-Azorín[2], Antonio Tristán-Vega[1], Rodrigo de Luis-García[1] and Santiago Aja-Fernández[1*]

[1]*Laboratorio de Procesado de Imagen (LPI), Universidad de Valladolid, Valladolid, Spain*
[2] *Headache Unit, Dept. of Neurology, Hospital Cl ´ınico Universitario de Valladolid, Spain*
[3] *Department of Medicine, Universidad de Valladolid, Valladolid, Spain*
[4] *Cardiff University Brain Research Imaging Centre (CUBRIC), School of Psychology, Cardiff University, Cardiff, UK*

A.- TBSS results

A.1. DTI measures

TBSS results for AD and MD are shown in Figure A1 and Figure A2, respectively. In addition, the statistically significant ROIs, according to the JHU-WM atlas, for each measure are included in Table A1 and Table A2 together with the minimum p-value obtained and the volume of each region.

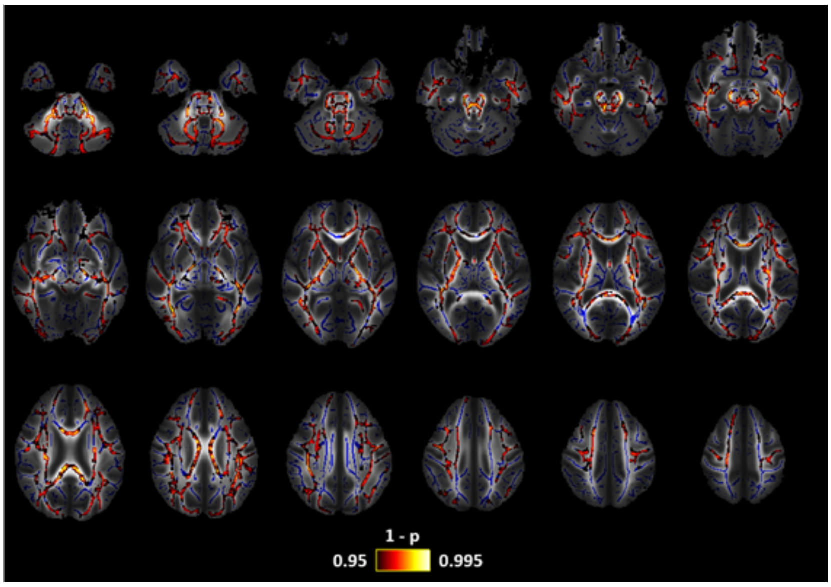

Figure A1. Axial Diffusivity (AD) alterations in patients with EM in comparison with CM. Lower AD values were found in CM. The white matter skeleton is shown in blue and voxels with significant differences in red-yellow. The color bar shows the 1-p values (family-wise error corrected).

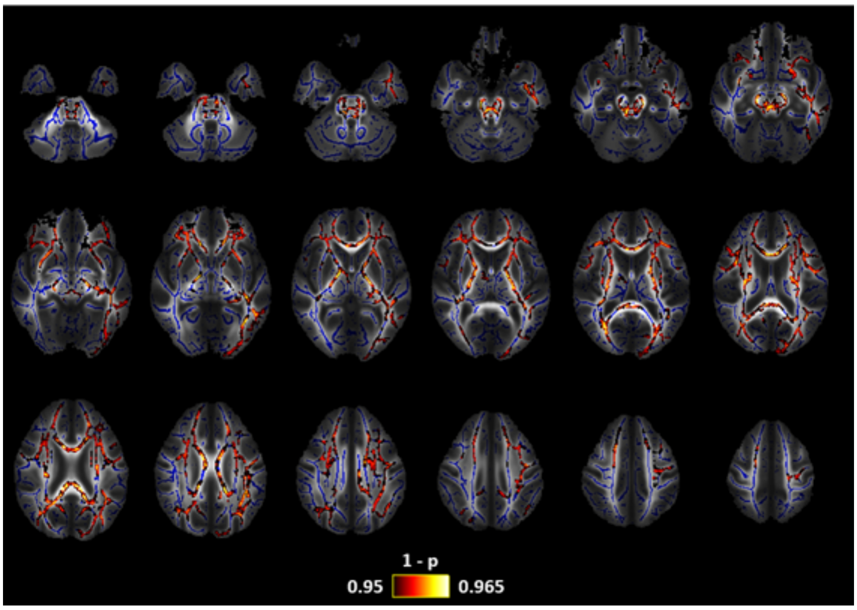

Figure A2. Mean Diffusivity (MD) alterations in patients with EM in comparison with CM. Lower MD values were found in CM. The white matter skeleton is shown in blue and voxels with significant differences in red-yellow. The color bar shows the 1-p values (family-wise error corrected).

Table A1. White matter regions from the ICBM-DTI-81 White Matter Atlas for which significant decreased AD values were found in CM compared to EM.

White Matter Region	Minimum *p*-Value (FWE-Corrected)	Volume (mm^3)
Middle cerebellar Peduncle	0.004	2175
Superior cerebellar peduncle R/L	0.0095/0.0095	142/122
Inferior cerebellar peduncle R/L	0.0094/0.005	67/74
Superior longitudinal fasciculus R/L	0.011/0.010	871/859
Genu of corpus callosum	0.012	435
Body of corpus callosum	0.013	856
Splenium of corpus callosum	0.016	785
Anterior corona radiata R/L	0.014/0.010	558/707
Superior corona radiata R/L	0.011/0.009	786/446
Posterior corona radiata R/L	0.013/0.009	148/226
External capsule R/L	0.010/0.009	437/541
Posterior limb of internal capsule R/L	0.011/0.009	598/577
Retrolenticular part of internal capsule R/L	0.012/0.009	443/334
Anterior limb of internal capsule R/L	0.011/0.010	270/268
Sagital Stratum R/L	0.012/0.009	460/356
Posterior thalamic radiation R/L	0.013/0.010	342/253
Cerebral peduncle R/L	0.008/0.009	227/263
Corticospinal tract R/L	0.008/0.009	110/169
Medial lemniscus R/L	0.009/0.008	89/114
Pontine crossing tract	0.008	77
Fornix (cres) R/L	0.013/0.010	91/57
Fornix (body and column)	0.036	44
Cingulum (hippocampus) L	0.0176	169
Tapetum R	0.013	34

Table A2. White matter regions from the ICBM-DTI-81 White Matter Atlas for which significant decreased MD values were found in CM compared to EM.

White Matter Region	Minimum *p*-Value (FWE-Corrected)	Volume (mm^3)
Middle cerebellar Peduncle	0.041	113
Superior cerebellar peduncle R/L	0.042/0.043	73/32
Superior fronto-occipital fasciculus R/L	0.038/0.037	49/64
Superior longitudinal fasciculus R/L	0.038/0.035	99/714
Genu of corpus callosum	0.041	782
Body of corpus callosum	0.040	948
Splenium of corpus callosum	0.038	966
Anterior corona radiata R/L	0.038/0.036	782/642
Superior corona radiata R/L	0.038/0.037	661/453
Posterior corona radiata R/L	0.038/0.035	100/58
External capsule R/L	0.040/0.036	340/444
Posterior limb of internal capsule R/L	0.037/0.038	509/507
Retrolenticular part of internal capsule R/L	0.046/0.041	113/202
Anterior limb of internal capsule R/L	0.038/0.036	275/363
Sagital Stratum L	0.040	229
Posterior thalamic radiation R/L	0.038/0.035	136/198
Cerebral peduncle R/L	0.044/0.040	165/208
Corticospinal tract R/L	0.044/0.040	95/144
Medial lemniscus R/L	0.043/0.044	56/47
Pontine crossing tract	0.043	130
Fornix (cres) R/L	0.046	122
Cingulum (cingulate gyrus) L	0.040	74
Tapetum R	0.040	35

A.2. AMURA measures

TBSS results for AMURA measures are displayed as follows: RTOP, Figure A3 and Table A3; RTAP, Figure A4 and Table A4; RTPP, Figure A5 and Table A5; qMSD, Figure A6 and Table A6; figures correspond to TBSS results and tables to the significant ROIs.

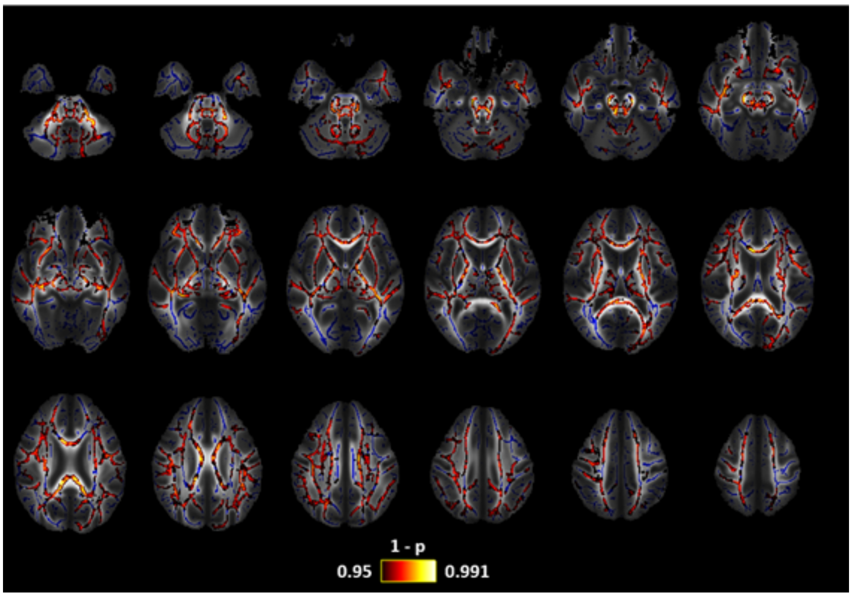

Figure A3. Return-to-origin (RTOP) alterations in patients with EM in comparison with HC. Lower RTOP values were found in EM. The white matter skeleton is shown in blue and voxels with significant differences in red-yellow. The color bar shows the 1-p values (family-wise error corrected).

Table A3. White matter regions from the ICBM-DTI-81 White Matter Atlas for which significantly decreased RTOP values were found in HC compared to EM.

White Matter Region	Minimum *p*-Value (FWE-Corrected)	Volume (mm^3)
Middle cerebellar Peduncle	0.0086	1663
Superior cerebellar peduncle R/L	0.086/0.0088	134/107
Inferior cerebellar peduncle R/L	0.0091/0.0099	132/93
Superior fronto-occipital fasciculus R/L	0.009/0.010	57/42
Superior longitudinal fasciculus R/L	0.012/0.010	858/705
Genu of corpus callosum	0.0094	801
Body of corpus callosum	0.012	1436
Splenium of corpus callosum	0.019	1592
Anterior corona radiata R/L	0.009/0.010	1085/661
Superior corona radiata R/L	0.009/0.010	852/561
Posterior corona radiata R/L	0.010/0.015	66/114
External capsule R/L	0.008/0.010	977/874
Posterior limb of internal capsule R/L	0.008/0.010	588/659
Retrolenticular part of internal capsule R/L	0.008/0.009	363/487
Anterior limb of internal capsule R/L	0.009/0.009	339/396
Sagital Stratum R/L	0.008/0.010	382/230
Posterior thalamic radiation R/L	0.021/0.016	52/245
Cerebral peduncle R/L	0.008/0.009	321/217
Corticospinal tract R/L	0.008/0.009	130/141
Medial lemniscus R/L	0.008/0.008	118/136
Pontine crossing tract	0.008	281
Fornix (cres) R/L	0.008/0.011	264/154
Uncinate fasciculus R/L	0.008/0.010	44/33

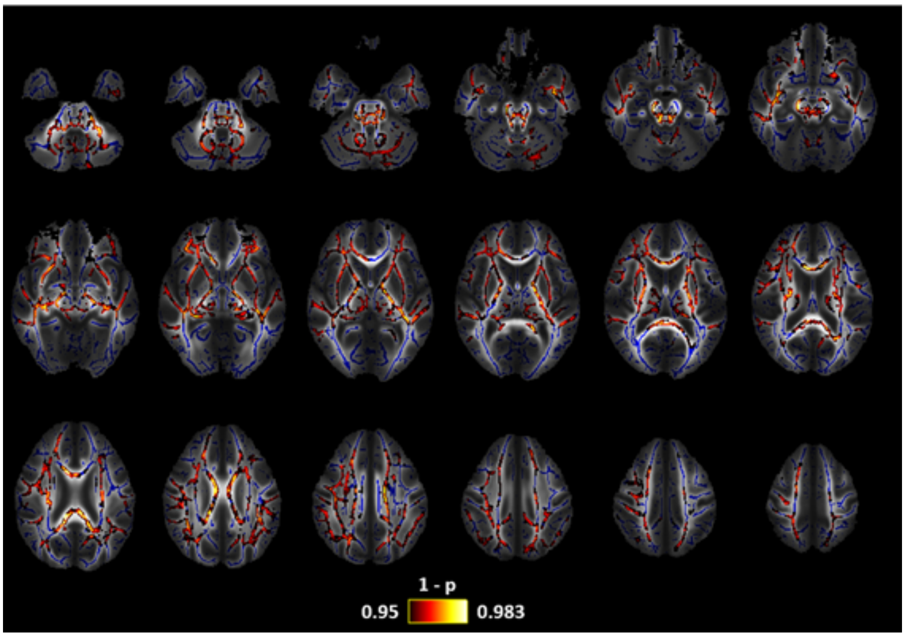

Figure A4. Return-to-axis (RTAP) alterations in patients with EM in comparison with HC. Lower RTAP values were found in EM. The white matter skeleton is shown in blue and voxels with significant differences in red-yellow. The color bar shows the 1-p values (family-wise error corrected).

Table A4. White matter regions from the ICBM-DTI-81 White Matter Atlas for which significant decreased RTAP values were found in HC compared to EM.

White Matter Region	Minimum *p*-Value (FWE-Corrected)	Volume (mm^3)
Middle cerebellar Peduncle	0.027	1131
Superior cerebellar peduncle R/L	0.025/0.026	91/83
Inferior cerebellar peduncle R/L	0.035/0.037	119/37
Superior fronto-occipital fasciculus R/L	0.022/0.034	296/42
Superior longitudinal fasciculus R/L	0.022/0.032	654/296
Genu of corpus callosum	0.019	459
Body of corpus callosum	0.029	1343
Splenium of corpus callosum	0.041	1164
Anterior corona radiata R/L	0.016/0.036	1031/395
Superior corona radiata R/L	0.020/0.035	668/4430
Posterior corona radiata R/L	0.041/0.039	53/69
External capsule R/L	0.018/0.035	921/759
Posterior limb of internal capsule R/L	0.025/0.033	486/615
Retrolenticular part of internal capsule R/L	0.022/0.033	190/387
Anterior limb of internal capsule R/L	0.021/0.036	318/413
Sagital Stratum R/L	0.022/0.035	298/136
Cerebral peduncle R/L	0.027/0.027	310/105
Corticospinal tract R/L	0.026/0.028	87/97
Medial lemniscus R/L	0.026/0.026	109/115
Pontine crossing tract	0.025	206
Fornix (cres) R/L	0.022/0.032	236/93
Uncinate fasciculus R/L	0.023/0.037	51/35

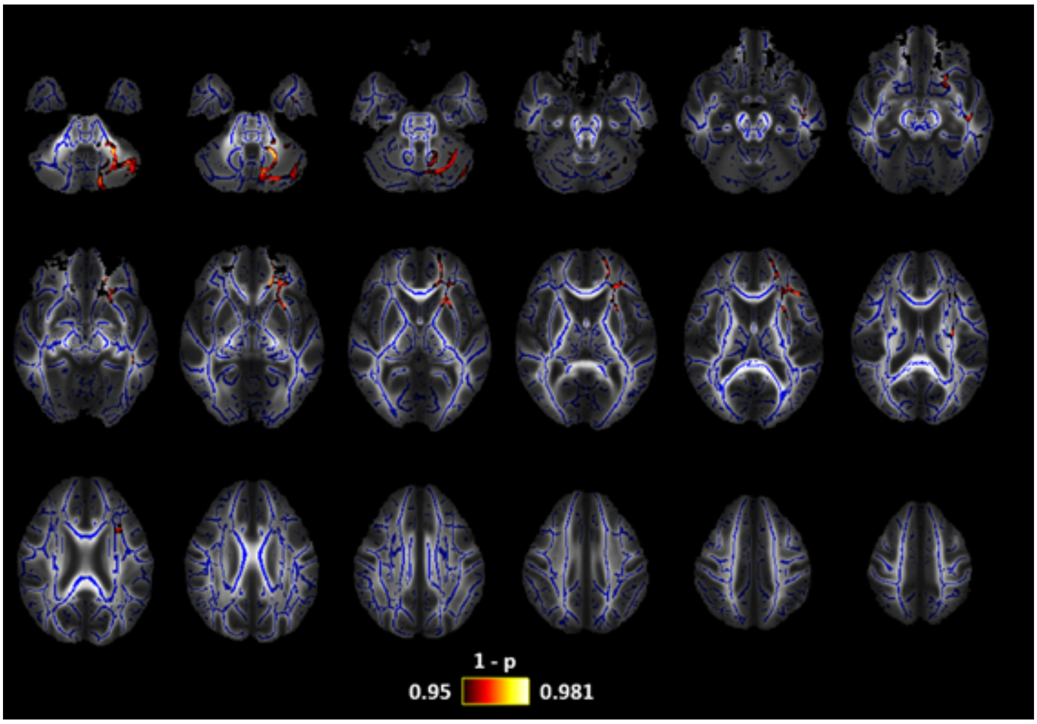

Figure A5. Return-to-plane (RTPP) alterations in patients with EM in comparison with HC. Lower RTPP values were found in EM. The white matter skeleton is shown in blue and voxels with significant differences in red-yellow. The color bar shows the 1-p values (family-wise error corrected).

Table A5. White matter regions from the ICBM-DTI-81 White Matter Atlas for which significant decreased RTPP values were found in HC compared to EM.

White Matter Region	Minimum *p*-Value (FWE-Corrected)	Volume (mm^3)
Middle cerebellar Peduncle	0.019	521
Anterior corona radiata L	0.041	251
External capsule L	0.045	145
Anterior limb of internal capsule R/L	0.045	45

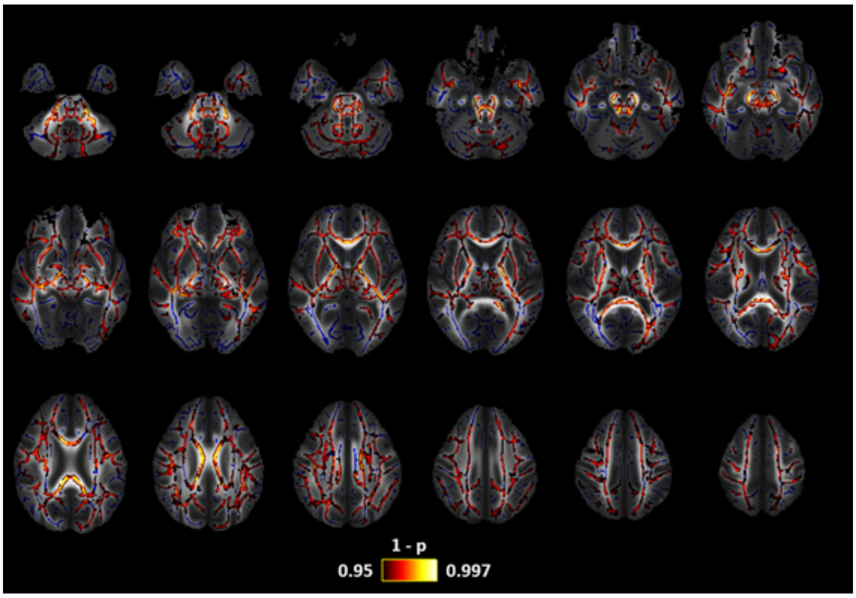

Figure A6. Q-Space Mean Square Displacement (qMSD) alterations in patients with EM in comparison with HC. Lower qMSD values were found in EM. The white matter skeleton is shown in blue and voxels with significant differences in red-yellow. The color bar shows the 1-p values (family-wise error corrected).

Table A6. White matter regions from the ICBM-DTI-81 White Matter Atlas for which significant decreased qMSD values were found in HC compared to EM.

White Matter Region	Minimum *p*-Value (FWE-Corrected)	Volume (mm^3)
Middle cerebellar Peduncle	0.002	2082
Superior cerebellar peduncle R/L	0.002/0.002	186/148
Inferior cerebellar peduncle R/L	0.002/0.003	168/137
Superior fronto-occipital fasciculus R/L	0.003/0.004	47/44
Superior longitudinal fasciculus R/L	0.004/0.004	919/799
Genu of corpus callosum	0.004	1077
Body of corpus callosum	0.005	1964
Splenium of corpus callosum	0.004	1891
Anterior corona radiata R/L	0.004/0.004	1142/649
Superior corona radiata R/L	0.004/0.003	866/637
Posterior corona radiata R/L	0.005/0.004	108/195
External capsule R/L	0.002/0.004	1083/958
Posterior limb of internal capsule R/L	0.002/0.004	717/744
Retrolenticular part of internal capsule R/L	0.002/0.004	417/502
Anterior limb of internal capsule R/L	0.003/0.004	397/463
Sagital Stratum R/L	0.002/0.004	397/226
Posterior thalamic radiation R/L	0.005/0.005	53/231
Cerebral peduncle R/L	0.002/0.003	487/400
Corticospinal tract R/L	0.002/0.003	170/209
Medial lemniscus R/L	0.002/0.002	159/154
Pontine crossing tract	0.002	316

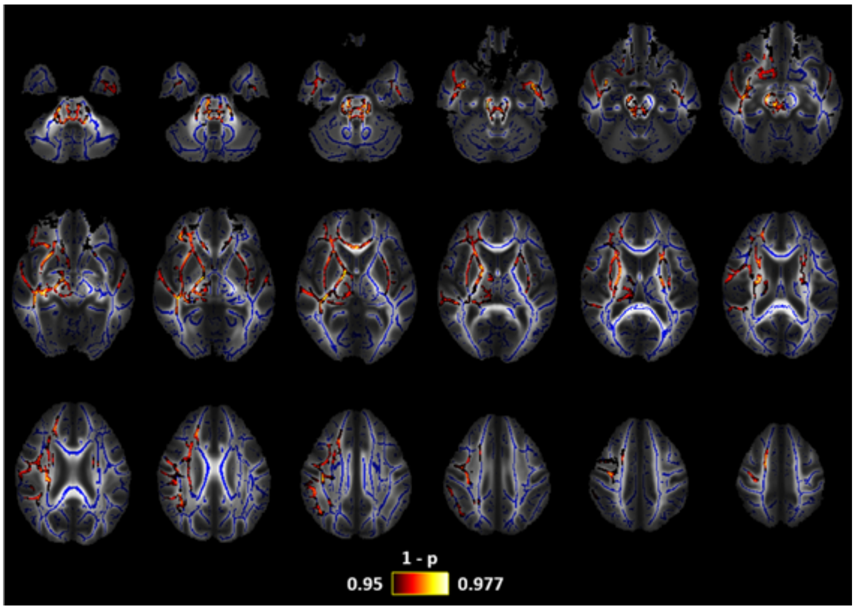

Figure A7. Propagator Anisotropy (PA) alterations in patients with EM in comparison with HC. Lower PA values were found in EM. The white matter skeleton is shown in blue and voxels with significant differences in red-yellow. The color bar shows the 1-p values (family-wise error corrected).

Table A7. White matter regions from the ICBM-DTI-81 White Matter Atlas for which significant decreased PA values were found in HC compared to EM.

White Matter Region	Minimum *p*-Value (FWE-Corrected)	Volume (mm^3)
Middle cerebellar Peduncle	0.038	333
Superior cerebellar peduncle R	0.041	44
Inferior cerebellar peduncle R/L	0.037/0.037	55/40
Superior fronto-occipital fasciculus R	0.025	88
Superior longitudinal fasciculus R	0.026	524
Genu of corpus callosum	0.038	206
Anterior corona radiata R/L	0.026/0.042	884/65
Superior corona radiata R	0.024	535
Posterior corona radiata R	0.036	42
External capsule R/L	0.022/0.044	973/327
Posterior limb of internal capsule R/L	0.024/0.049	474/65
Retrolenticular part of internal capsule R	0.035	229
Anterior limb of internal capsule R/L	0.025/0.046	438/87
Sagital Stratum R	0.036	206
Cerebral peduncle R	0.027	207
Corticospinal tract R/L	0.036/0.040	230/136
Medial lemniscus R/L	0.037/0.037	130/109
Pontine crossing tract	0.037	210
Fornix (cres) R	0.034	145
Uncinate fasciculus R	0.038	64

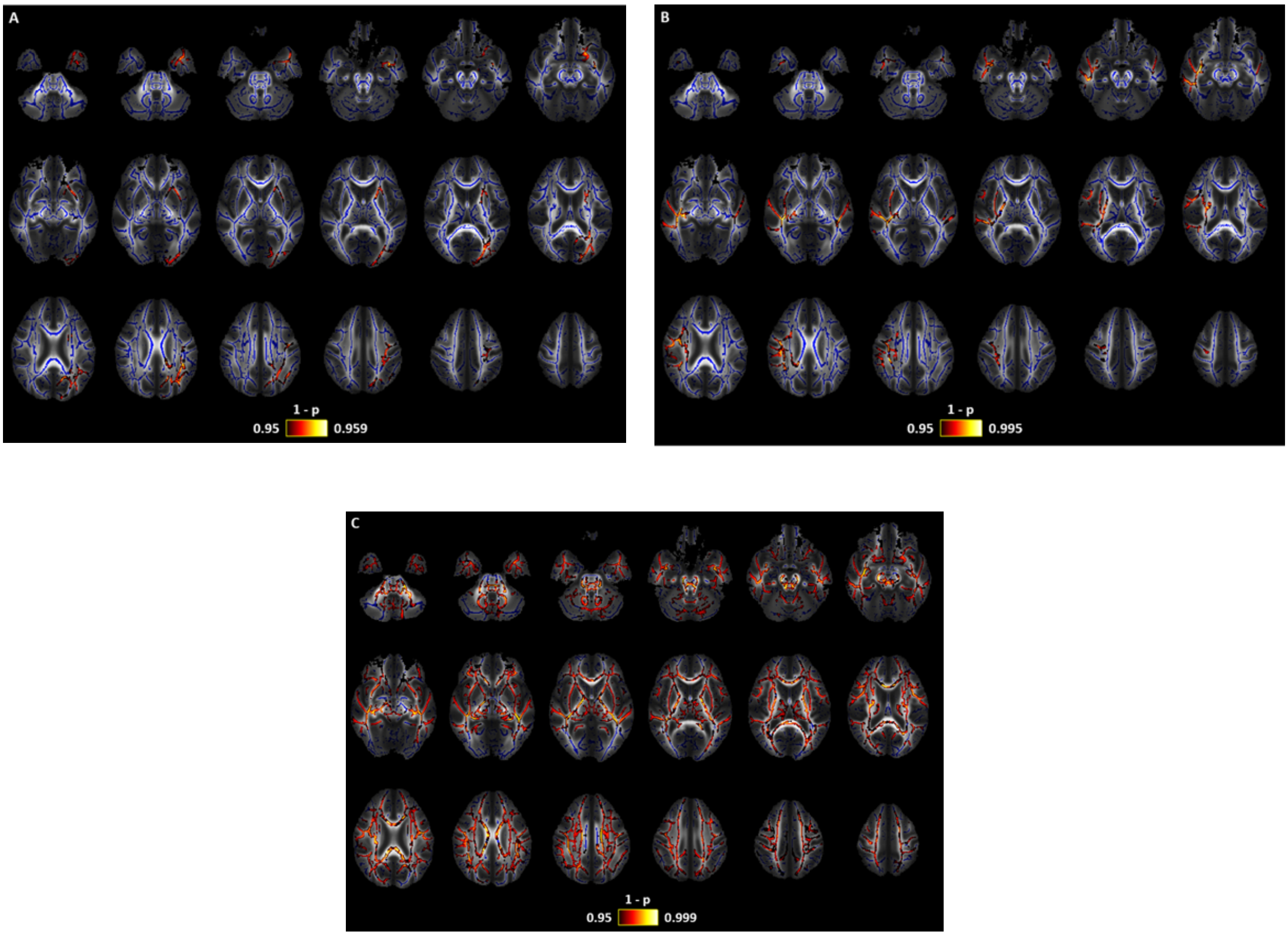

Figure A8. Non-Gaussianity (DDT) alterations. A) Patients with CM in comparison with EM. Lower NG values were found in EM. B) HC in comparison with CM patients. Lower NG values were found in CM. C) HC in comparison with EM patients. Lower NG values were found in EM. The white matter skeleton is shown in blue and voxels with significant differences in red-yellow. The color bar shows the 1-p values (family-wise error corrected).

Table A8. White matter regions from the ICBM-DTI-81 White Matter Atlas for which significant decreased DDT values were found in HC compared to CM, HC compared to EM and CM compared to EM.

	**CM compared to EM**	
**White Matter Region**	**Minimum *p*-Value (FWE-Corrected)**	**Volume (mm³)**
Splenium of corpus callosum	0.042	77
Superior longitudinal fasciculus L	0.042	281
Superior fronto-occipital fasciculus L	0.048	49
Anterior limb of internal capsule L	0.048	189
Posterior thalamic radiation L	0.042	98
External capsule L	0.046	341
Tapelum L	0.049	66
	**HC compared to CM**	
Splenium of corpus callosum	0.037	43
Superior corona radiata R	0.017	318
Posterior corona radiata R		
External capsule R	0.008	686
Posterior limb of internal capsule R	0.017	245
Retrolenticular part of internal capsule R	0.006	352
Sagital Stratum R	0.006	381
Fornix (cres) R	0.009	109
	**HC compared to EM**	
Middle cerebellar Peduncle	0.0004	1680
Superior cerebellar peduncle R/L	0.0004/0.0004	125/115
Inferior cerebellar peduncle R/L	0.001/0.001	152/91
Superior fronto-occipital fasciculus R/L	0.0002/0.0008	91/66
Superior longitudinal fasciculus R/L	0.0002/0.0004	1382/1114
Genu of corpus callosum	0.0016	783
Body of corpus callosum	0.0006	1950
Splenium of corpus callosum	0.0008	1397
Anterior corona radiata R/L	0.0004/0.0004	1124/891
Superior corona radiata R/L	0.0002/0.0006	1296/1080
Posterior corona radiata R/L	0.0002/0.001	512/346
External capsule R/L	0.0002/0.0004	1357/1217
Posterior limb of internal capsule R/L	0.0002/0.0004	669/643
Retrolenticular part of internal capsule R/L	0.0002/0.0002	535/624
Anterior limb of internal capsule R/L	0.0002/0.0004	391/489
Sagital Stratum R/L	0.0002/0.0002	501/424
Posterior thalamic radiation R/L	0.0002/0.0006	254/219
Cerebral peduncle R/L	0.0004/0.0008	355/150
Corticospinal tract R/L	0.0004/0.0006	106/144
Medial lemniscus R/L	0.0004/0.0006	131/154
Pontine crossing tract	0.0004	310
Fornix (cres) R/L	0.0002/0.0004	310/260
Cingulum (cingulate gyrus) L	0.0024	100
Cingulum (hippocampus) R	0.005	198
Tapetum R/L	0.005/0.008	60/38
Uncinate fasciculus R/L	0.0002/0.0002	60/49

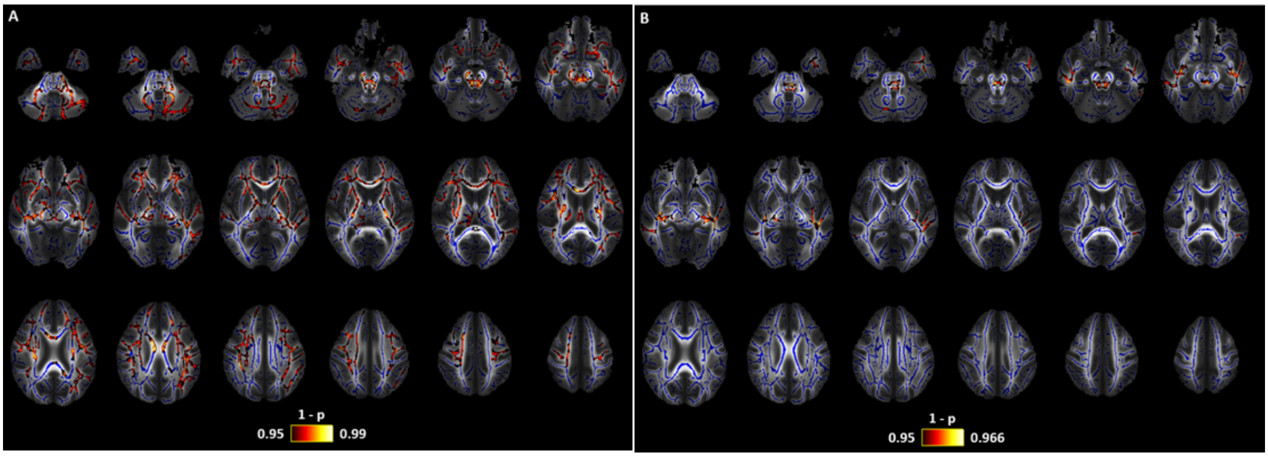

Figure A9. Moment 1/2 of the Ensemble Average Propagator (MUA) alterations. A) Patients with CM in comparison with EM. Lower MUA values were found in EM. B) HC in comparison with EM patients. Lower NG values were found in EM. The white matter skeleton is shown in blue and voxels with significant differences in red-yellow. The color bar shows the 1-p values (family-wise error corrected).

Table A9. White matter regions from the ICBM-DTI-81 White Matter Atlas for which significant decreased MUA values were found in CM compared to EM and HC compared to EM.

	**HC compared to EM**	
**White Matter Region**	**Minimum *p*-Value (FWE-Corrected)**	**Volume (mm^3)**
External capsule R/L	0.044/0.035	80/179
Retrolenticular part of internal capsule L	0.039	39
Sagital Stratum R/L	0.041/0.036	113/118
Medial lemniscus L	0.043	44
Pontine crossing tract	0.042	107
Fornix (cres) R/L	0.045/0.036	35/74
	**CM compared to EM**	
Middle cerebellar Peduncle	0.009	1324
Superior cerebellar peduncle R/L	0.029/0.029	54/97
Inferior cerebellar peduncle L	0.013	46
Superior fronto-occipital fasciculus R	0.024	37
Superior longitudinal fasciculus R/L	0.023/0.023	694/734
Genu of corpus callosum	0.020	488
Body of corpus callosum	0.023	548
Splenium of corpus callosum	0.048	66
Anterior corona radiata R/L	0.038/0.020	488/630
Superior corona radiata R/L	0.023/0.023	750/330
Posterior corona radiata R/L	0.041/0.033	55/56
External capsule R/L	0.023/0.021	478/615
Posterior limb of internal capsule R/L	0.023/0.026	443/271
Retrolenticular part of internal capsule L	0.028	205
Anterior limb of internal capsule R/L	0.027/0.021	297/188
Sagital Stratum R/L	0.034/0.025	152/227
Cerebral peduncle R	0.032	238
Corticospinal tract R/L	0.032	36
Medial lemniscus R/L	0.033	72
Pontine crossing tract	0.029	87
Fornix (cres) R/L	0.035/0.029	103/91
Fornix (column and body)	0.041	41

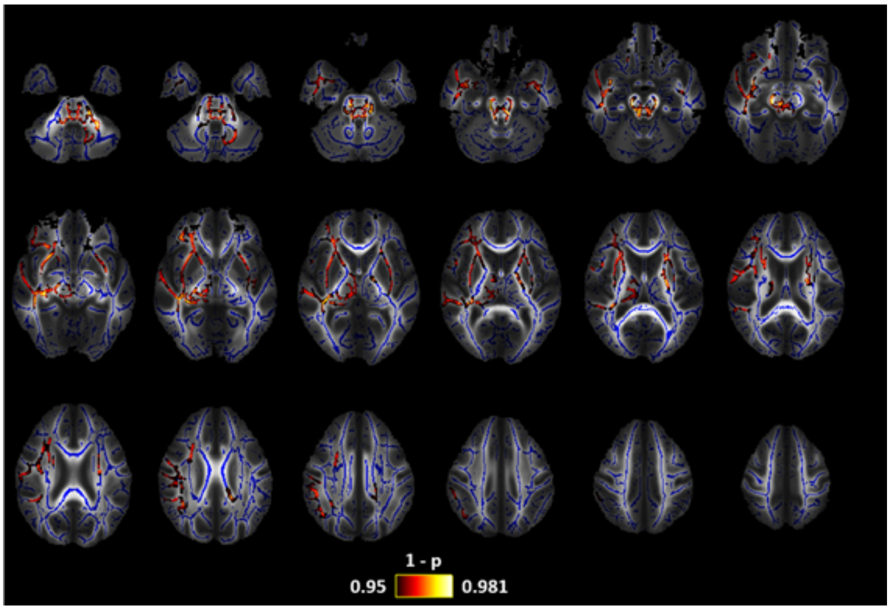

Figure A10. Diffusion Anisotropy (DiA) alterations in patients with EM in comparison with HC. Lower DiA values were found in EM. The white matter skeleton is shown in blue and voxels with significant differences in red-yellow. The color bar shows the 1-p values (family-wise error corrected).

Table A10. White matter regions from the ICBM-DTI-81 White Matter Atlas for which significant decreased DiA values were found in EM compared to HC.

White Matter Region	Minimum *p*-Value (FWE-Corrected)	Volume (mm^3)
Middle cerebellar Peduncle	0.020	546
Superior cerebellar peduncle R/L	0.020/0.021	94/78
Inferior cerebellar peduncle R/L	0.019/0.020	72/43
Superior fronto-occipital fasciculus R	0.020	64
Superior longitudinal fasciculus R	0.019	368
Body of corpus callosum	0.044	38
Splenium of corpus callosum	0.044	59
Anterior corona radiata R/L	0.019/0.042	695/53
Superior corona radiata R/L	0.020/0.045	248/95
External capsule R/L	0.018/0.042	956/382
Posterior limb of internal capsule L	0.042	206
Retrolenticular part of internal capsule R	0.019	320
Anterior limb of internal capsule R/L	0.019/0.042	242/137
Sagital Stratum R	0.019	265
Cerebral peduncle R/L	0.019/0.024	243/74
Corticospinal tract R/L	0.019/0.019	224/160
Medial lemniscus R/L	0.018/0.019	148/132
Pontine crossing tract	0.020	154
Fornix (cres) R	0.019	193
Uncinate fasciculus R	0.022	62

B.- Definition of AMURA metrics used in the study

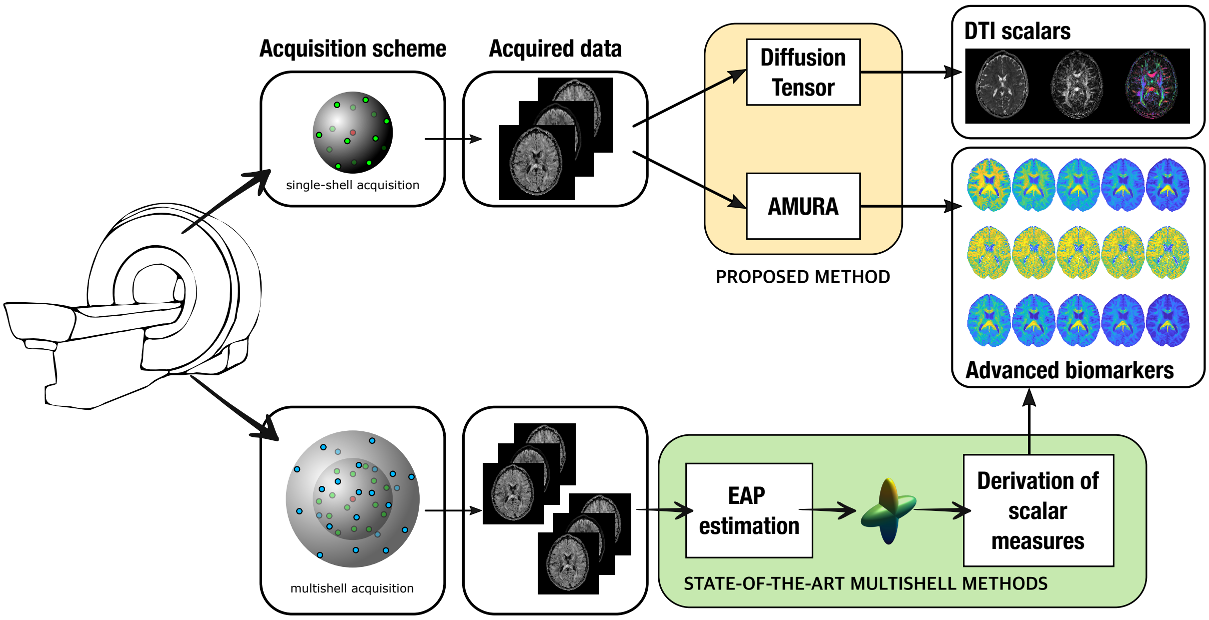

AMURA allows the estimation of the EAP-related scalars without the explicit calculation of the EAP, using a lower number of samples, even with a single-shell acquisition scheme. AMURA considers that, if the amount of data is reduced, a restricted diffusion model consistent with single-shell acquisitions must be assumed: the ADC $D(\boldsymbol{q})$ does not depend on the radial direction (i.e., on the magnitude of the q-vector) within the range of b-values probed, so that $D(\boldsymbol{q}) = D(\boldsymbol{u})$ where $\boldsymbol{u}$ is a unit direction in space where $\|\boldsymbol{u}\| = 1$ and $\boldsymbol{q} = q\boldsymbol{u}$. This way, assuming a general Gaussian diffusion profile, the normalized magnitude image provided by the MRI scanner, $E(\boldsymbol{q})$, becomes:

$$E(\boldsymbol{q}) = \exp\left(-4\pi^2\tau q^2 D(\boldsymbol{u})\right) = \exp\left(-b \cdot D(\boldsymbol{u})\right)$$

From this equation, AMURA proposed a particular implementation of scalar measures. Since the mono-exponential model only holds within a limited range around the measured b-value, the measures derived this way must be seen as *apparent* values at a given b-value, related to the original ones but dependent on the selected shell. The AMURA metrics used in the paper are the following ones:

RTOP	$RTOP = \frac{1}{(4\pi)^2\tau^{3/2}} C_{0,0}\left\{D(\boldsymbol{u})^{-3/2}\right\}$
RTPP	$RTPP = \frac{1}{(4\pi\tau)^{1/2}} \left(D(r_0)\right)^{-1/2}$
RTAP	$RTAP = \frac{1}{8\pi^2\tau} \mathcal{G}\{D(\boldsymbol{u})^{-1}\}(r_0)$

PA	$$PA = \gamma\left(\left(1-\frac{4}{\sqrt{\pi}}\frac{\left[C_{0,0}\left\{(D(\boldsymbol{u})+D_{AV})^{-\frac{3}{2}}\right\}\right]^2}{C_{0,0}\left\{\left(D(\boldsymbol{u})\right)^{-\frac{3}{2}}\right\}D_{AV}^{-\frac{3}{2}}}\right)^{\frac{1}{2}}\right)$$
DiA	$$DiA = \left(1-\frac{1}{\sqrt{4\pi}}\frac{C_{0,0}^2\{D(\boldsymbol{u})\}}{C_{0,0}\{D^2(\boldsymbol{u})\}}\right)^{\frac{1}{2}}$$
QMSD	$$qMSD = \Gamma\left(\frac{5}{2}\right)\frac{\sqrt{\pi}}{(4\pi^2\tau)^{5/2}}\ C_{0,0}\left\{D(\boldsymbol{u})^{-5/2}\right\}$$
$\Upsilon^{1/2}$	$$\Upsilon^{1/2} = \Gamma\left(\frac{3+1/2}{2}\right)\frac{\sqrt{\pi}}{(4\pi^2\tau)^{(3+\frac{1}{2})/2}}\ C_{0,0}\left\{D(\boldsymbol{u})^{-(3+\frac{1}{2})/2}\right\}$$
DDT	$$DDT = \left(1-2^3\frac{\left[C_{0,0}\left\{(D(\boldsymbol{u})+D_{DT}(\boldsymbol{u}))^{-\frac{3}{2}}\right\}\right]^2}{C_{0,0}\left\{\left(D(\boldsymbol{u})\right)^{-\frac{3}{2}}\right\}\ C_{0,0}\left\{\left(D_{DT}(\boldsymbol{u})\right)^{-\frac{3}{2}}\right\}}\right)^{\frac{1}{2}}$$

Notation:

- $D(\boldsymbol{u}) = -\frac{1}{\mathrm{b}}\log E(\boldsymbol{q})$
- $C_{0,0}\{H(\boldsymbol{u})\}$ DC component of signal $H(\boldsymbol{u})$ calculated using the zero-th order coefficient of a Spherical Harmonics (SH) expansion.
- r_0 direction of maximal diffusion.
- $\mathcal{G}\{H(\boldsymbol{u})\}(r_0)$ Funk-Radon Transform of $H(\boldsymbol{u})$ evaluated in the direction of maximal diffusion r_0
- $D_{AV} = \frac{1}{\sqrt{4\pi}}C_{0,0}\{D(\boldsymbol{u})\}$ average diffusivity.
- $D_{DT}(\boldsymbol{u})$ Diffusion Tensor equivalent of $D(\boldsymbol{u})$ (SH approximation of the diffusivity $D(\boldsymbol{u})$ using only two coefficients).
